# Predicting the Potential Distribution of Rare and Endangered *Emmenopterys henryi* in China Under Climate Change

**DOI:** 10.1002/ece3.70403

**Published:** 2024-10-13

**Authors:** Hanwei Cai, Guangfu Zhang

**Affiliations:** ^1^ Jiangsu Key Laboratory of Biodiversity and Biotechnology, School of Life Sciences Nanjing Normal University Nanjing China

**Keywords:** *Emmenopterys henryi*, environmental variables, global change, population centroid, suitable range

## Abstract

Climate change has a pivotal impact on the potential distribution of endangered and relic tree species. Probably due to unrepresentative sampling and single algorithm, at present, there are different views on the potential range of the endangered tree *Emmenopterys henryi,* endemic to China. Here, we first collated 612 occurrence records and 22 environmental variables including climate, topography, and soil. Combined the Biomod2 with MaxEnt, we then predicted its past, current, and future potential suitable area in China, and determined the key factors influencing its distribution. The ensemble model results showed that the main environmental variables affecting this species were the minimum temperature of the coldest month (BIO6), precipitation of warmest quarter (BIO18), and temperature seasonality (BIO4). Its current potential distribution area was 176.53 × 10^4^ km^2^, mainly concentrated in eastern, central, and southwestern China. Collectively, the suitable area of *E. henryi* would averagely decrease by 3.90% in all 16 future scenarios, with its centroid largely migrating northeastward. Our findings indicate that the endangered *E. henryi* covered 18 provinces in China, having a larger area than known. Moreover, climate change may have an adverse effect on its potential distribution. In addition, the ensemble model can produce more effective prediction outcomes than MaxEnt for such endemic tree species with large environmental range. We recommend increasing sample representativeness by analyzing the completeness properties of sample coverage, and simultaneously selecting appropriate algorithms to ensure the reliability of distribution prediction for endangered and relict tree species.

## Graphical abstract

We applied Biomod2 and MaxEnt to predict the current and future potential distribution of endangered *Emmenopterys henryi* in China. The current potential distribution area from Biomod2 is 176.53 × 10^4^ km^2^, which is larger than from MaxEnt. Moreover, climate change is expected to have an adverse effect on its potential distribution.

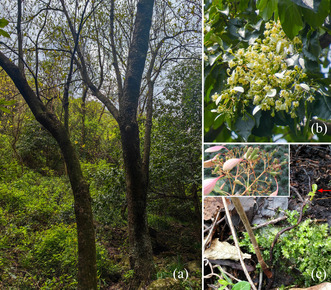

## 1 Introduction

Climate change has a profound effect on the growth and distribution of trees worldwide (Zhou, Lu, and Zhang 2023). Such an impact may be more severe for endangered tree species, which tend to have smaller population size, more restricted distribution, and lower genetic diversity relative to widespread species (Zhang et al. 2022). Accordingly, they are less tolerant to climate change than common tree species. Since the Fourth Industrial Revolution, growing global warming has possibly exacerbated habitat fragmentation for many species, and even led to their habitat loss to a great extent (Ye et al. 2021). As a result, this renders some of them on the verge of extinction (Vincent et al. 2020). Indeed, climate change has taken a great impact on the geographical distribution of endemic and endangered tree species over the past decades (Abolmaali, Tarkesh, and Bashari 2018; Yan and Zhang 2022). Therefore, it is of great significance for such endemic trees to determine their geographical shift in response to climate change, which contributes to developing appropriate conservation strategies.

**FIGURE 1:**
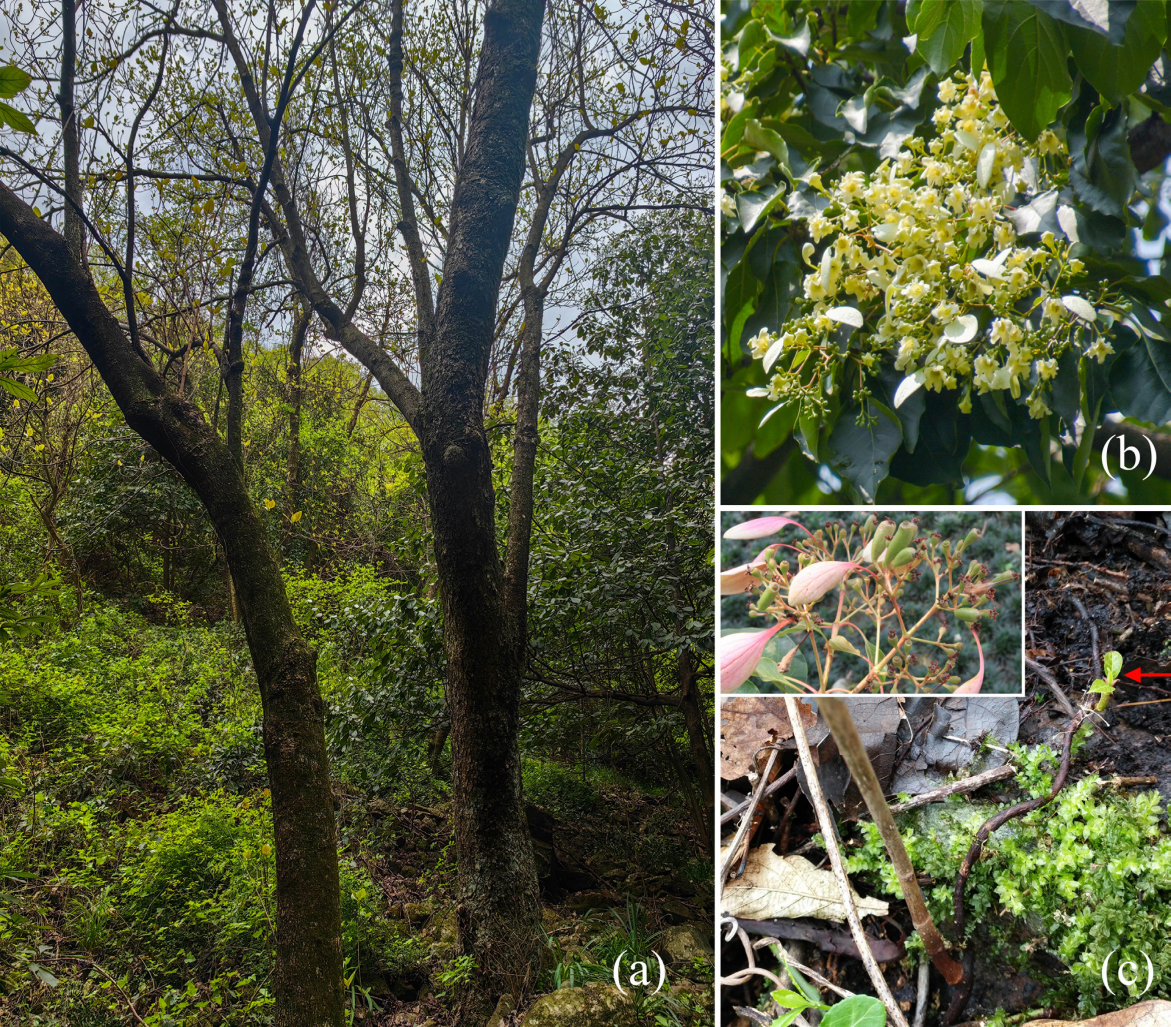
Photos of *Emmenopterys henryi* in the field. (a) Individuals in valley; (b) Inflorescence of *E. henryi*; and (c) Sprouting of *E. henryi*. Capsules with reddish bracts at the top left. The photos were taken by Guangfu Zhang.

*Emmenopterys henryi* Oliver is a deciduous tree from the Rubiaceae family. It is the only extant species of the monospecific genus *Emmenopterys* (Wu and Raven 2011), and a tertiary relict tree (Zhang et al. 2016; Shang, Song, and Da 2016). Following the methodology provided by Wu et al. in 2006, this genus falls into the areal‐type of endemic to China. Floristically, the endemic genus belongs to the subtype “Endemic to eastern and central China” (Hao 1997). As a tall tree, *E. henryi* has peculiar and fragrant flowers in shape since one of its outer sepals turns into a leaf‐like bract and is persistent on its fruit. Therefore, it is considered an excellent ornamental tree species (Figure 1). Meanwhile, it is a precious timber tree species with straight texture, fine structure, and uniformity wood (Zhang 2020). In addition, it can be utilized for the production of waxed paper and rayon cotton because of the soft and fine bark fibers. As early as 1992, it was listed as a “rare species” in *China Plant Red Data Book: Rare and Endangered Plants* (Fu 1992). Due to population decreasing and range shrinking resulting from human interferences, this species was decreed a second‐class national protected plant and listed on the checklist of *The Important Wild Plants for Conservation in China* in 2021 (Zhang and Yi 2023). Furthermore, it was classified as a “nearly threatened” (NT) species in the IUCN Red List (Qin 2020).

*Emmenopterys henryi* is distributed in 14 provinces of China, according to *Flora of China* (Chinese version). In light of *Flora of China* (English version), this species occurs in 15 provinces (Wu and Raven 2011). More recently, this species was extensively sampled from 14 provinces in China to reveal its genetic diversity (Zhang et al. 2016). In addition, it is reported that *E. henryi* is also distributed in Shaanxi province (Fan et al. 2012). As a whole, existing studies have shown that *E. henryi* is distributed in 17 provinces in China. Namely, they are Anhui, Chongqing, Fujian, Gansu, Guangdong, Guangxi, Guizhou, Henan, Hubei, Hunan, Jiangsu, Jiangxi, Shaanxi, Shanxi, Sichuan, Yunnan, and Zhejiang. Nevertheless, this tree usually has isolated small populations or naturally patchy habitats in most provinces (Zhang and Yi 2023). In recent years, some researchers have applied the species distribution model to address the potential distribution of *E. henryi* and its main influencing factors (Chen et al. 2022; Tang et al. 2023).

Species distribution models (SDMs) can serve as an instrument to simulate the potential distribution of species based on their occurrence records and related environmental variables (Heikkinen et al. 2006; Chen et al. 2020). Compared with mechanistic models, nowadays correlative models have been increasingly used to reveal species' suitable habitat because of the accessibility of these data. This is especially true when predicting the response of species to climate change (Du et al. 2021), searching for wild populations of endangered species (Wang, Zhi, and Zhang 2024), and assessing effects of habitat fragmentation on species distribution (Dong et al. 2024). At present, there are distinctive correlative models based on various algorithms, including generalized additive model (GAM), maximum entropy model (MaxEnt), generalized linear model (GLM), multivariate adaptive regression spline model (MARS), random forest (RF) model, ecological niche factor analysis model (ENFA), artificial neural network (ANN) model, and genetic algorithm rule‐set production model (GARP) (Sillero et al. 2021). Among them, MaxEnt is one of the most widely used SDMs (Merow, Smith, and Silander 2013; Abdelaal et al. 2019). And it has been successfully applied to predict the geographic distribution of endangered plants and identify the main environmental factors affecting their distribution under climate change scenarios (Dhyani et al. 2021).

In general, each model has its own advantages and disadvantages. As a result, an ensemble model has been developed by integrating various correlative models to improve the predicted outcomes. The Biomod2 model is a reliable multi‐model ensemble prediction platform. There are 10 species distribution models in Biomod2 package, namely, ANN, FDA (Flexible Discriminant Analysis), GAM, CTA (Classification Tree Analysis), GBM (Generalized Boosted Model), GLM, SRE (Surface Range Envelope), MARS, MaxEnt, and RF. More recently, it has taken to forecast suitability of habitats for endangered species (Xu et al. 2022; Huang et al. 2023).

Currently, there are different outcomes regarding *E. henryi*'s geographical distribution forecasted by MaxEnt. Chen et al. (2022) used 156 occurrence records in southwestern China and three types of variables (i.e., bioclimate, topography, and soil) to predict the distribution of *E. henryi*. They considered that its suitable area was 11.05 × 10^4^ km^2^, and that T‐clay was identified as the most important factor. Besides, its suitable area would increase slightly under future climate scenarios. In contrast, Tang et al. (2023) used 122 occurrence records and 19 bioclimatic variables to forecast the potential distribution of this species in China. They pointed out that the suitable area was 137.64 × 10^4^ km^2^, which is much larger than that obtained by Chen et al. (2022). The most important variable was the mean temperature of the driest quarter (BIO9), which is quite distinct from Chen et al. (2022). Meanwhile, they further considered that its suitable areas would decrease in the future. The major reasons for such differences may result from the poor sampling and single algorithm. Besides, both only performed individual models, namely MaxEnt.

Here, according to field surveys and literature collection, we conducted both ensemble modeling and MaxEnt modeling to determine the suitable area of *E. henryi*. The objective of this study is to reveal its geographical range and its response to climate change. Specifically, we aim to address the following questions: (1) What are the main factors affecting the potential distribution of *E. henryi*? (2) What is the current suitable area of *E. henryi*? (3) How will the geographical distribution shift for *E. henryi* in future scenarios? (4) Which one is better? MaxEnt or Biomod2 for *E. henryi*?

## 2 Materials and Methods

### 2.1 Species Occurrence Records

Occurrence records of *E. henryi* were obtained from field surveys, websites, and related literatures. (1) Investigating in the field from 2021 to 2023, we carried out comprehensive surveys for *E. henryi* wild populations in Anhui, Jiangsu, and Zhejiang, and other provinces in East China to acquire their spatial localities; (2) searching through resource sharing platforms, which include the Global Biodiversity Information Facility (GBIF, https://www.gbif.org/), the National Specimen Information Infrastructure (NSII, http://nsii.org.cn/), the Chinese Virtual Herbarium (CVH, https://www.cvh.ac.cn/), and the Plant Photo Bank of China (PPBC, http://ppbc.iplant.cn/); (3) consulting published literatures and relevant reports. We used the key words of *E. henryi*'s specific name and Latin name in *Flora of China*, provincial floras, papers containing its localities and related reports to gather its occurrence records.

For individual data with specific distribution information but no corresponding coordinates, we used Google Maps to determine latitude and longitude data, which were accurate to two decimal places. After that, we removed such data as cultivated (i.e., botanical gardens, schools, and parks) plants or those that could not be determined to specific locations. Lastly, we collected 798 natural occurrence records of *E. henryi*.

**FIGURE 2:**
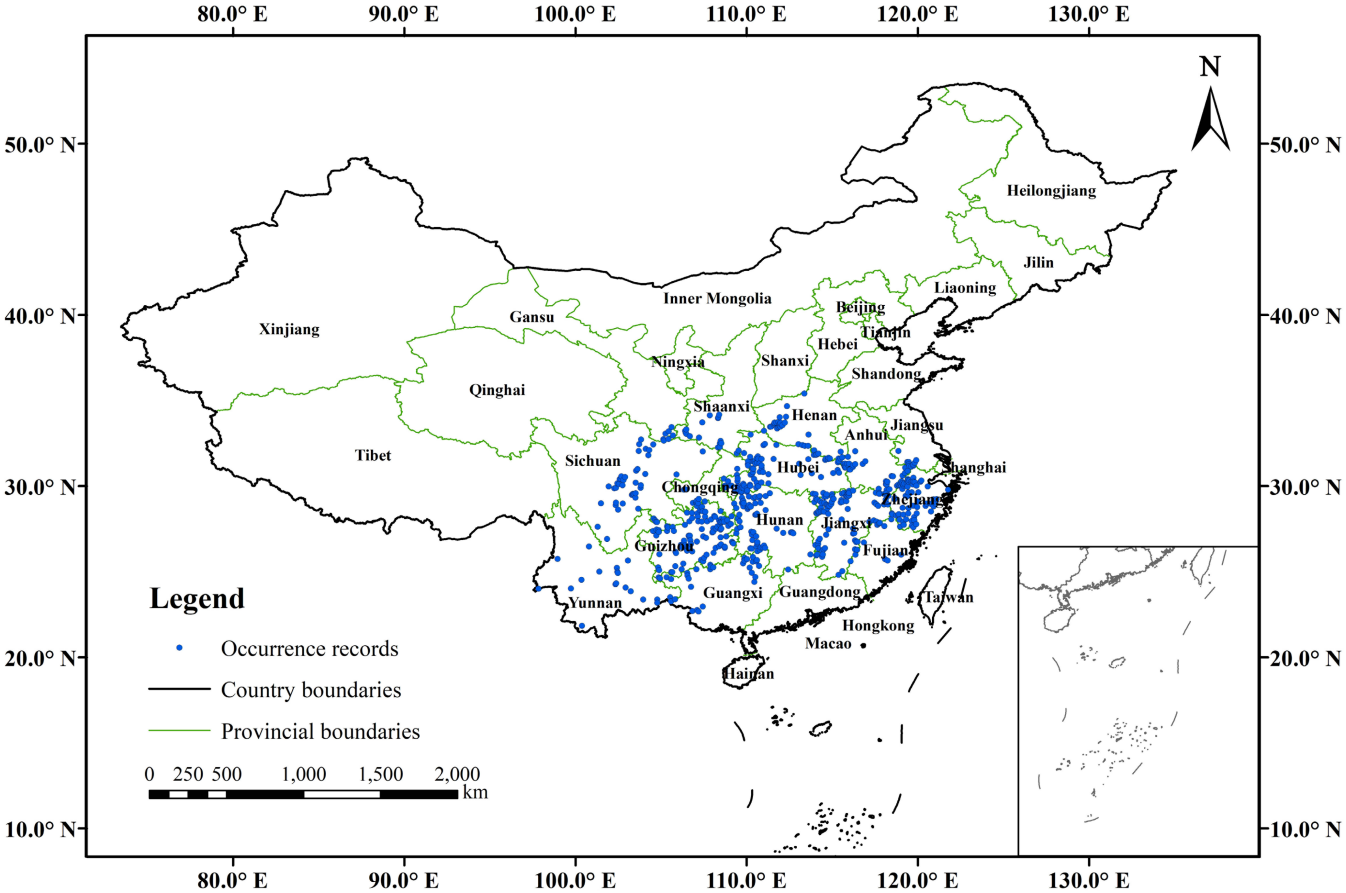
Current occurrence of *Emmenopterys henryi* in China.

After eliminating erroneous and duplicate records, we employed the tool of “Spatially‐Rarefy‐Occurrence‐Data” for SDMs in the SDMtoolbox to ensure that each 5 × 5 km grid contained only one occurrence record (Brown and Anderson 2014; Zhou, Lu, and Zhang 2023). This approach aims to minimize the number of occurrence records existing spatial autocorrelation and prevent overfitting (Brown, Bennett, and French 2017). Finally, we acquired 612 occurrence records of *E. henryi* (Figure 2) and saved them in a “.csv” file for modeling (Table S1).

### 2.2 Environmental Variables

Three types of environmental variables were used in this study. (1) Topographic data: elevation data were downloaded from the WorldClim database (https://www.worldclim.org/) and slope data were extracted from the DEM database (http://www.tuxingis.com). (2) Climate data: 19 bioclimatic variables were downloaded from the WorldClim database, including past, current, and future climate data. The past climate data were based on the periods of the Last Glacial Maximum and the Middle Holocene. The current bioclimatic variables are based on climatic conditions during 1970–2000 (WorldClim V2.1). The future climate data are based on the BCC‐CSM2‐MR global climate model since this model is more suitable for Asia, especially for China (Yang, Jiang, and Li 2016; Shi et al. 2020). The future climate data include four shared socioeconomic pathways (SSP1‐2.6, SSP2‐4.5, SSP3‐7.0, and SSP5‐8.5, which represent different pathways of climate change, ranging from the lowest to the highest emission scenarios) under four time periods (2021–2040, 2041–2060, 2061–2080, and 2081–2100). The spatial resolution of the climate data for *E. henryi* is 2.5 min (~ 5 km) because of its large sample size and range‐wide distribution. Furthermore, some studies have shown that the prediction results of 2.5 min data and 30 s data have similar accuracy, and the calculation speed of 2.5 min data is faster than that of 30 s (Guisan et al. 2007; Wan et al. 2021). (3) Soil data: soil datasets based on the Harmonized World Soil Database (HWSD) (V1.2) (http://www.tpdc.ac.cn/zh‐hans/) were downloaded from the Tibetan Plateau National Data Center, and 16 topsoil data (0–30 cm) were selected.

In order to prevent the model from overfitting, the collected environmental variables need to remove the multicollinearity. Firstly, ArcMap V10.8 was used to process all the environmental variables into a uniform coordinate system, resolution size, and spatial extent. We then converted the “.tif” format to “.asc” format (Gao et al. 2021; Ma et al. 2021). Secondly, ensemble modeling was employed to simulate the bioclimatic, soil, and topography variables initially to obtain the importance percentage of the 38 environmental variables. Finally, the “Remove‐Highly‐Correlated‐Variables” tool in SDMtoolbox 2.0 was utilized to rank these variables in terms of their importance. The Pearson correlation coefficient is more widely used to reduce collinearity in predictor sets than the other methods like variance inflation factor (VIF), and principal component analysis (PCA) (Brun et al. 2020). The maximum correlated coefficient was set to 0.8. When the absolute value of Pearson correlation coefficient between two environmental variables is greater than 0.8, a variable with a greater contribution to modeling is reserved for subsequent analysis (Brown, Bennett, and French 2017; Feng et al. 2019). Finally, a total of 22 variables, including 8 bioclimatic, 2 topography, and 12 soil ones, were selected to build the final model.

### 2.3 Model Calibration

We used MaxEnt (V3.4.4) and Biomod2 (V3.5.1) to simulate the current distribution of *E. henryi*, respectively. First, we optimized the MaxEnt model. Generally, there are three factors affecting the complexity of the MaxEnt model, which include the number of environmental variables, FC (Features Class), and RM (Regularization Multiplier) (Kong, Li, and Zou 2019). The operations of the MaxEnt model consist of six different combinations of FC (L, LQ, H, LQH, LQHP, and LQHPT, where L = linear features, Q = quadratic features, H = hinge features, P = product features, and T = threshold features). Similarly, the RM of the MaxEnt model takes values in the range 0.5–4 (with an interval of 0.5), with a total of eight values (Muscarella et al. 2014). We used the ENMeval 2.0.4 installation package in R4.2.1 to calculate and select the optimal combination of operating parameters for the MaxEnt model (Phillips, Anderson, and Schapire 2006). When delta. AICc = 0, parameter combinations are the optimal (Warren and Seifert 2011; Chen et al. 2020). Therefore, the optimal parameter combination for this study is FC = LQH and RM = 1.5. In addition, 75% of the occurrence records were randomly selected as the training set and the remaining 25% as the test set. The operation was repeated 10 times to ensure the predictive accuracy of the model.

Second, we generated an ensemble model using the Biomod2 package. Combined with 22 variables, the 612 occurrence records of *E. henryi* were first performed in the Biomod2 package with each of the 10 models. Then, we obtained its values of AUC (area under curve) and TSS (true skill statistics) for each model. The models with AUC > 0.8 and TSS > 0.7 (Zhao 2018) among the 10 models were selected for creating an ensemble model. As a result, the ensemble model was consisted of nine individual models, namely ANN, CTA, FDA, GAM, GBM, GLM, MARS, MaxEnt, and RF. When the number of pseudo‐absences is nearly equal to the number of species' distribution points, the models with classification and machine‐learning techniques are able to obtain accurate predicted distributions (Barbet‐Massin et al. 2012; Liu, Graeme, and Matt 2019). Afterward, 1000 pseudo‐absence points were randomly generated by R during the modeling process (Chang et al. 2024). Moreover, 75% of the occurrence records were randomly selected as the training set and the remaining 25% as the test set. To ensure the prediction accuracy of the model, the operation was repeated 10 times to obtain the average value as the final modeling result.

### 2.4 Geospatial Data Analysis

The outcomes of the MaxEnt and ensemble models were visualized using ArcMap 10.8, respectively. The classification of model results was based on the “Maximum test sensitivity and specificity thresholds”, which are considered best practices for presence‐only models (Baldwin 2009; Liu, White, and Newell 2013). Accordingly, habitat suitability was categorized into four classes: unsuitable area (0–0.20), low suitable area (0.20–0.47), moderately suitable area (0.47–0.73), and highly suitable area (0.73–1.00) (Srivastava, Griess, and Keena 2020; Ren et al. 2020; Xu et al. 2021). Meanwhile, the range of moderately (0.47–0.73) and highly suitable area (0.73–1) is considered to be suitable area for the growth of *E. henryi* (Yan and Zhang 2022). In order to determine the effect of climate change on the distribution of *E. henryi*, the suitable area for each type was calculated separately. Meanwhile, ArcMap10.8 was used to calculate the centroid of the suitable area under different climate scenarios, and vector files of the direction and centroid change of the suitable area in different periods were generated to express the direction and distance of the centroid migration under different climate scenarios (Liang et al. 2018; Wang, Zhi, and Zhang 2024).

## 3 Results

### 3.1 Model Performance

We performed 10 individual models using Biomod2 for *E. henryi* with 612 occurrence records and 22 environmental variables. Nine of these models had AUC > 0.8 and TSS > 0.7 except the SRE. Therefore, they were selected to build an ensemble model. The prediction accuracy of each model (both individual and ensemble models) was shown in Table 1. Except for ANN or CTA, which was very close to 0.9 in terms of AUC, the other seven models had the AUC values greater than 0.9, indicating that these models have reached an excellent level. In addition, the nine models had the TSS values greater than 0.7, indicating that they had high credibility and accuracy. For example, MaxEnt had the AUC value of 0.9240 and TSS value of 0.7897, and GBM model had the highest AUC of 0.9428 and the highest TSS of 0.7982. In contrast, the ensemble model had the AUC and TSS values of 0.9715 and 0.8405, respectively, and both were higher than each of the nine individual models. Accordingly, this indicated that the ensemble model of *E. henryi* performed much better than the individual model.

**TABLE 1:** The mean value of the area under curve (AUC) and true skill statistic (TSS) for each modeling algorithm and the ensemble model (in bold) of *Emmenopterys henryi*.

Model	AUC		TSS	
	Mean	SD	Mean	SD
Artificial neural network (ANN)	0.8872	0.0160	0.7389	0.0260
Classification tree analysis (CTA)	0.8942	0.0169	0.7694	0.0306
Flexible discriminant analysis (FDA)	0.9364	0.0109	0.7883	0.0355
Generalized additive model (GAM)	0.9247	0.0082	0.7787	0.0257
Generalized boosted model (GBM)	0.9428	0.0084	0.7982	0.0313
Generalized linear model (GLM)	0.9376	0.0083	0.7841	0.0298
Multivariate adaptive regression spline (MARS)	0.9361	0.0076	0.7771	0.0330
Maximum entropy (MAXENT)	0.9240	0.0007	0.7897	0.0021
Random forest (RF)	0.9415	0.0419	0.7903	0.0419
**Ensemble model**	**0.9715**	**0.0035**	**0.8405**	**0.0007**

Therefore, the ensemble model was applied to forecast the current and future *E. henryi* distribution. Meanwhile, MaxEnt was also used to predict its current distribution in our analysis to compare our results with those from previous studies.

### 3.2 Main Environmental Variables Affecting *E. henryi*

Among the three types of environmental variables, the contribution percentages of bioclimatic, topography, and soil variables were 92.95%, 5.84%, and 1.20%, respectively (Table 2). The contribution of bioclimatic variables was nearly 16 times that of topography ones. This indicated that climate had a greater influence on the potential distribution of *E. henryi* compared with the other two factors.

**TABLE 2:** Percentage of importance of different environmental variables in the current scenario for *Emmenopterys henryi*.

Type of variables	Code	Environmental variables	Contribution percentage (%)
Bioclimate	BIO6	Min temperature of coldest month	51.12
	BIO18	Precipitation of warmest quarter	19.88
	BIO4	Temperature seasonality (standard deviation × 100)	9.29
	BIO3	Isothermal	6.63
	BIO15	Precipitation seasonality (coefficient of variation)	2.43
	BIO2	Mean diurnal range [Mean of monthly (max temp‐min temp)]	1.77
	BIO10	Mean temperature of warmest quarter	1.61
	BIO14	Precipitation of driest month	0.22
Topography	ELE	Elevation	5.78
	EXP	Exposure	0.06
Soil	T_ESP	Top layer exchangeable sodium salt	0.30
	T_BS	Top layer basic saturation	0.18
	T_USDA_TEX	Top layer soil texture classification	0.12
	T_CACO_3_	Top layer carbonate or lime content	0.10
	T_OC	Top layer organic carbon content	0.09
	T_CEC_CLAY	Top clay layer soil cation exchange capacity	0.08
	T_SILT	Top layer powder sand particle content	0.07
	T_TEB	Top layer soil exchangeable base	0.06
	T_CEC_SOIL	Top layer soil cation exchange capacity	0.06
	T_GRAVEL	Top layer gravel volume percentage	0.06
	T_CLAY	Top layer clay content	0.05
	T_ECE	Top layer electrical conductivity	0.03

The ensemble model also showed that among the selected eight bioclimatic variables, the top three were BIO6 (min temperature of the coldest month) (51.12%), BIO18 (precipitation of warmest quarter) (19.88%), and BIO4 (temperature seasonality) (9.29%), respectively, and the sum of their contribution percentage was more than 80%. Therefore, they were identified as the three key bioclimatic factors influencing the potential distribution of *E. henryi*.

Response curves were used to determine the relationship between environmental variables and the probability of species occurrence, reflecting the biological tolerance and habitat suitability (Abolmaali, Tarkesh, and Bashari 2018). According to the classification of suitable area, when the probability of species presence is greater than 0.47, it indicates that the environmental conditions were suitable for the growth of *E. henryi*.

**FIGURE 3:**
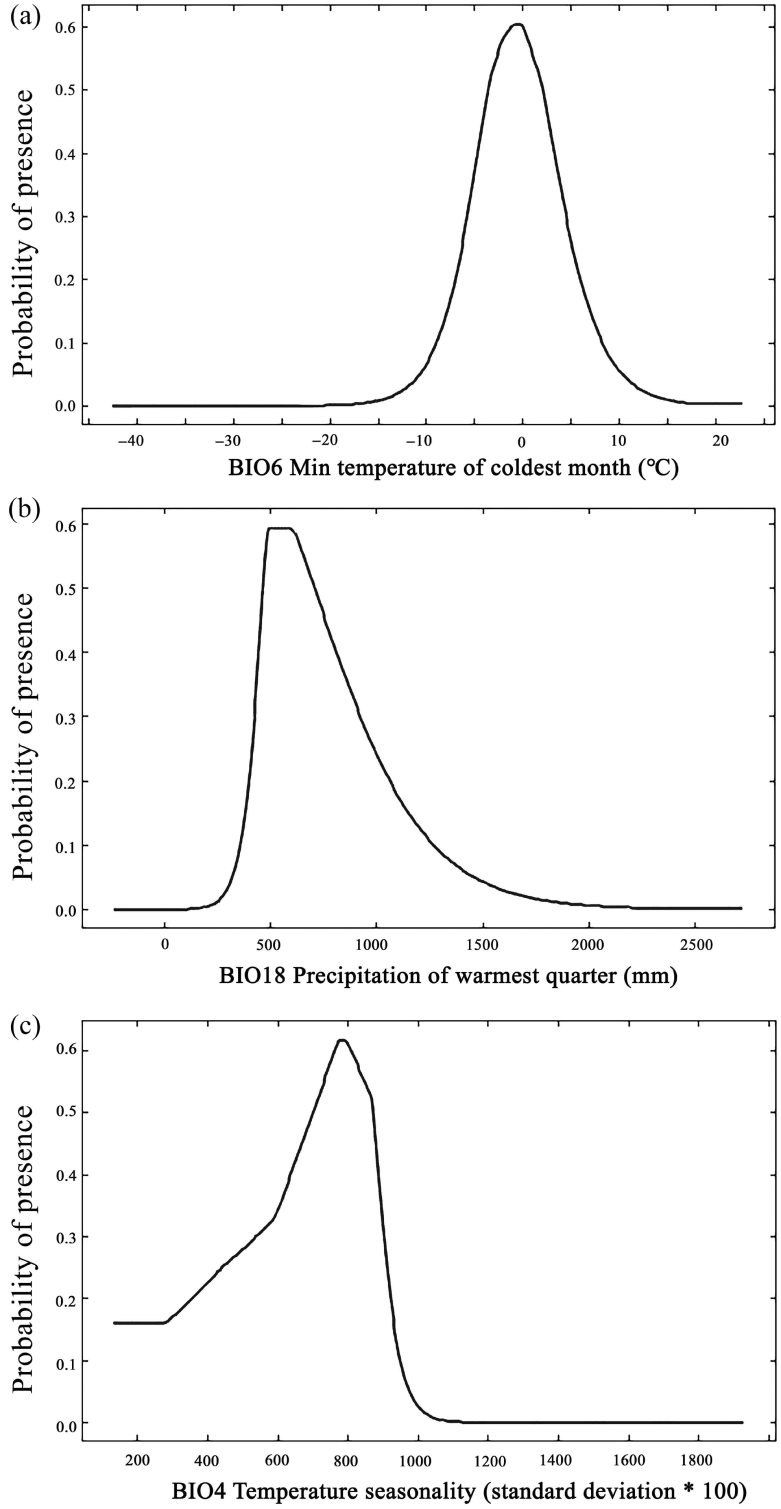
Response curves of *Emmenopterys henryi* to major environment variables. (a) BIO6: min temperature of coldest month; (b) BIO18: precipitation of warmest quarter; and (c) BIO4: temperature seasonality.

The presence probability of *E. henryi* showed a trend of first increasing and then decreasing in the minimum temperature of the coldest month (BIO6) (Figure 3a). It was optimal for *E. henryi* when BIO6 ranged from −4°C to 2°C with the maximum of 1°C. The precipitation of the warmest quarter (BIO18) ranged from 450 to 700 mm, which was suitable for the growth of *E. henryi*, and the presence probability reached the maximum at 500 mm (Figure 3b). When temperature seasonality (BIO4) was between 700 and 850, the presence probability of *E. henryi* exceeded 0.47, reaching the maximum at 800 (Figure 3c).

### 3.3 Current Geographical Distribution of *E. henryi*

**FIGURE 4:**
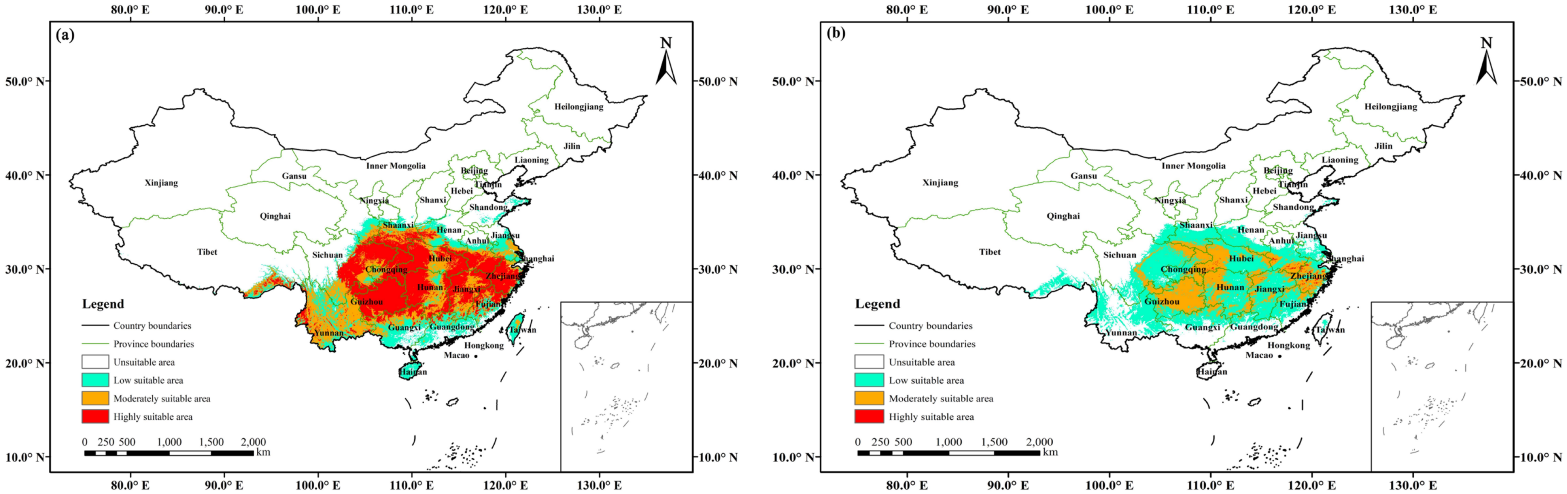
Suitable area of *Emmenopterys henryi* predicted by ensemble models (a) and by MaxEnt (b) in the current scenario.

Currently, the results of the ensemble model showed that the suitable area (i.e., moderately and highly) of *E. henryi* was located in 19 provinces, such as Anhui, Jiangsu, Jiangxi, Zhejiang in East China, Hunan, Hubei, Henan in Central China, and Sichuan, Guizhou, Yunnan and other provinces in southwestern China (Figure 4a). Their total suitable area amounted to 176.53 × 10^4^ km^2^, accounting for 18.48% of China's total territory; the highly suitable area covered 102.40 × 10^4^ km^2^, accounting for 10.72%. Likewise, MaxEnt modeling predicted that the suitable area of *E. henryi* was mainly distributed in eastern, central, and southwestern China, which included 15 provinces in total, such as Zhejiang, Hubei, and Guizhou provinces (Figure 4b). The total suitable area covered 60.30 × 10^4^ km^2^, accounting for 6.31% of China; the highly suitable area was 1.05 × 10^4^ km^2^, accounting for only 0.11%.

Notably, the suitable area predicted by the MaxEnt model was significantly smaller than that predicted by the ensemble model. Namely, the result from MaxEnt was approximately one‐third of that from the ensemble model in a suitable area, with only 1% in highly suitable area.

### 3.4 Past and Future Geographical Distribution Shift of *E. henryi*

**FIGURE 5:**
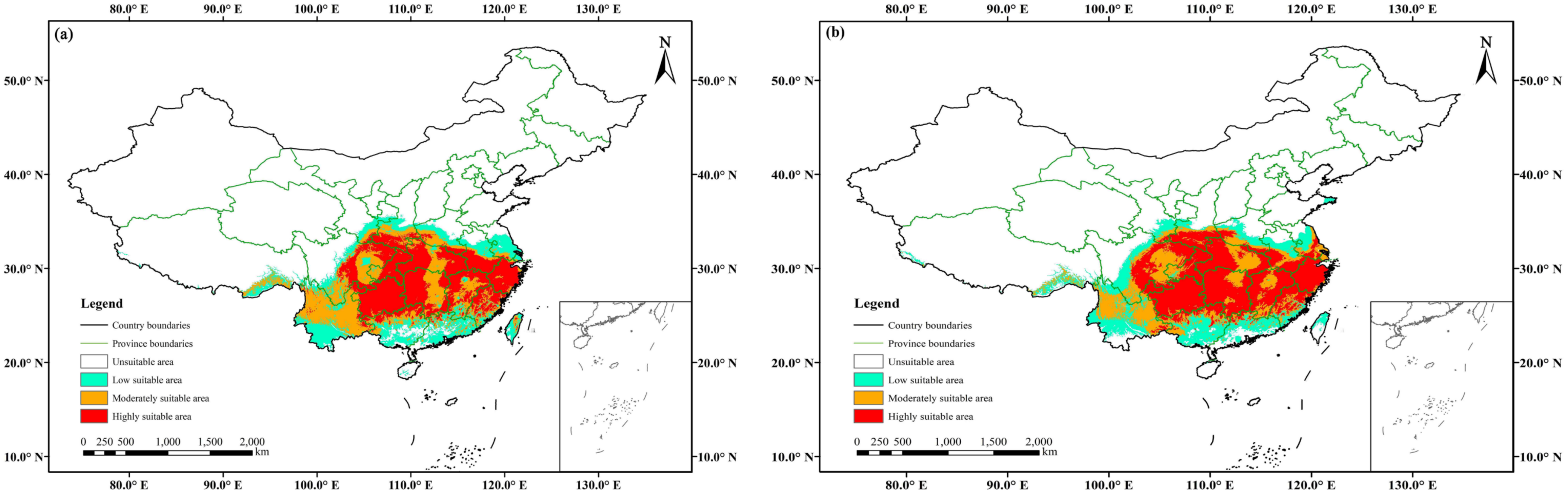
Prediction of changes in the suitable area of *Emmenopterys henryi* in the past scenarios by ensemble models. (a) The Last Glacial Maximum (LGM) and (b) the Middle Holocene (MH).

In the past, during the Last Glacial Maximum, suitable areas for *E. henryi* were primarily concentrated in eastern China (i.e., southern Anhui, Zhejiang, and northern Jiangxi), central China (i.e., western Hubei, Hunan), and southwestern China. (i.e., eastern Sichuan, Chongqing and Guizhou) (Figure 5a). The total suitable area amounted to 172.99 × 10^4^ km^2^, accounting for 18.11% of China's total territory, which is smaller than the current suitable area (Table 3).

**TABLE 3:** Percentage and trend of potential suitable area for *Emmenopterys henryi* under different climate scenarios.

Scenarios	Low suitable		Moderately suitable		Highly suitable		Suitable area (moderately and highly)	
	Percentage of area (%)	Trend (%)	Percentage of area (%)	Trend (%)	Percentage of area (%)	Trend (%)	Percentage of area (%)	Trend (%)
LGM	6.30	+ 3.45	7.93	+ 2.19	10.18	− 5.04	18.11	− 2.00
MID	6.16	+ 1.15	6.66	− 14.18	11.45	+ 6.81	18.12	− 1.95
Current	6.09	—	7.76	—	10.72	—	18.48	—
2021–2040								
SSP12.6	7.12	16.91	6.26	− 19.33	11.43	6.62	17.69	− 4.27
SSP2‐4.5	6.49	6.57	6.34	− 18.3	11.75	9.61	18.09	− 2.11
SSP3‐7.0	6.01	− 1.32	6.95	− 10.44	10.89	1.59	17.84	− 3.46
SSP5‐8.5	6.4	5.09	6.46	− 16.75	10.78	0.56	17.24	− 6.71
$\bar{x}\pm \mathrm{SD}$	6.51 ± 0.46	6.90 ± 7.55	6.50 ± 0.31	− 16.24 ± 3.99	11.21 ± 0.46	4.57 ± 4.26	17.715 ± 0.36	− 4.14 ± 1.93
2041–2060								
SSP1‐2.6	6.09	0	5.93	− 23.58	11.25	4.94	17.18	− 7.03
SSP2‐4.5	7.23	18.72	6.08	− 21.65	11.64	8.58	17.72	− 4.11
SSP3‐7.0	6.66	9.36	6.73	− 13.27	11.69	9.05	18.42	− 0.32
SSP5‐8.5	7.01	15.11	7.37	− 5.026	10.88	1.49	18.25	− 1.24
$\bar{x}\pm \mathrm{SD}$	6.75 ± 0.50	10.84 ± 8.17	6.53 ± 0.66	− 15.85 ± 8.51	11.37 ± 0.38	6.06 ± 3.53	17.89 ± 0.56	− 3.19 ± 3.03
2061–2080								
SSP1‐2.6	6.41	5.25	6.43	− 17.14	11.48	7.09	17.91	− 3.08
SSP2‐4.5	6.34	4.11	6.47	− 16.62	11.02	2.8	17.49	− 5.36
SSP3‐7.0	7	14.94	5.9	− 23.97	11.94	11.38	17.84	− 3.46
SSP5‐8.5	6.32	3.78	6.54	− 15.72	10.72	0	17.26	− 6.6
$\bar{x}\pm \mathrm{SD}$	6.52 ± 0.23	7.06 ± 5.32	6.34 ± 0.29	− 18.3 ± 3.78	11.29 ± 0.53	5.32 ± 4.98	17.625 ± 0.30	− 4.63 ± 1.65
2081–2100								
SSP1‐2.6	8.15	33.83	6.4	− 17.53	10.77	0.47	17.17	− 7.09
SSP2‐4.5	6.21	1.97	7.22	− 6.96	10.45	− 2.52	17.67	− 4.38
SSP3‐7.0	6.67	9.52	7.56	− 2.58	10.65	− 0.65	18.21	− 1.46
SSP5‐8.5	6.08	− 0.16	6.78	− 12.63	11.4	6.34	18.18	− 1.62
$\bar{x}\pm \mathrm{SD}$	6.78 ± 0.95	11.33 ± 15.59	6.99 ± 0.51	− 9.92 ± 6.53	10.82 ± 0.41	0.93 ± 3.82	17.81 ± 0.49	− 3.63 ± 2.66
Total $\bar{x}\pm \mathrm{SD}$	6.64 ± 0.56	8.99 ± 9.14	6.59 ± 0.49	− 15.09 ± 6.29	11.17 ± 0.46	4.21 ± 4.26	17.76 ± 0.41	− 3.90 ± 2.21

In the Middle Holocene, suitable habitats for *E. henryi* were similar to last Glacial Maximum, which were mainly distributed in eastern China (i.e., southern Jiangsu, southern Anhui, Zhejiang, and northern Jiangxi), central China (i.e., western Hubei, Hunan) and southwestern China. (i.e., eastern Sichuan, Chongqing, and Guizhou) (Figure 5b). Compared with the Last Glacial Maximum, the suitable distribution in the Middle Holocene exhibited a more continuous pattern with less fragmentation. The total suitable area amounted to 173.09 × 10^4^ km^2^, showing a little increase compared to the last Glacial Maximum (Table 3).

**FIGURE 6:**
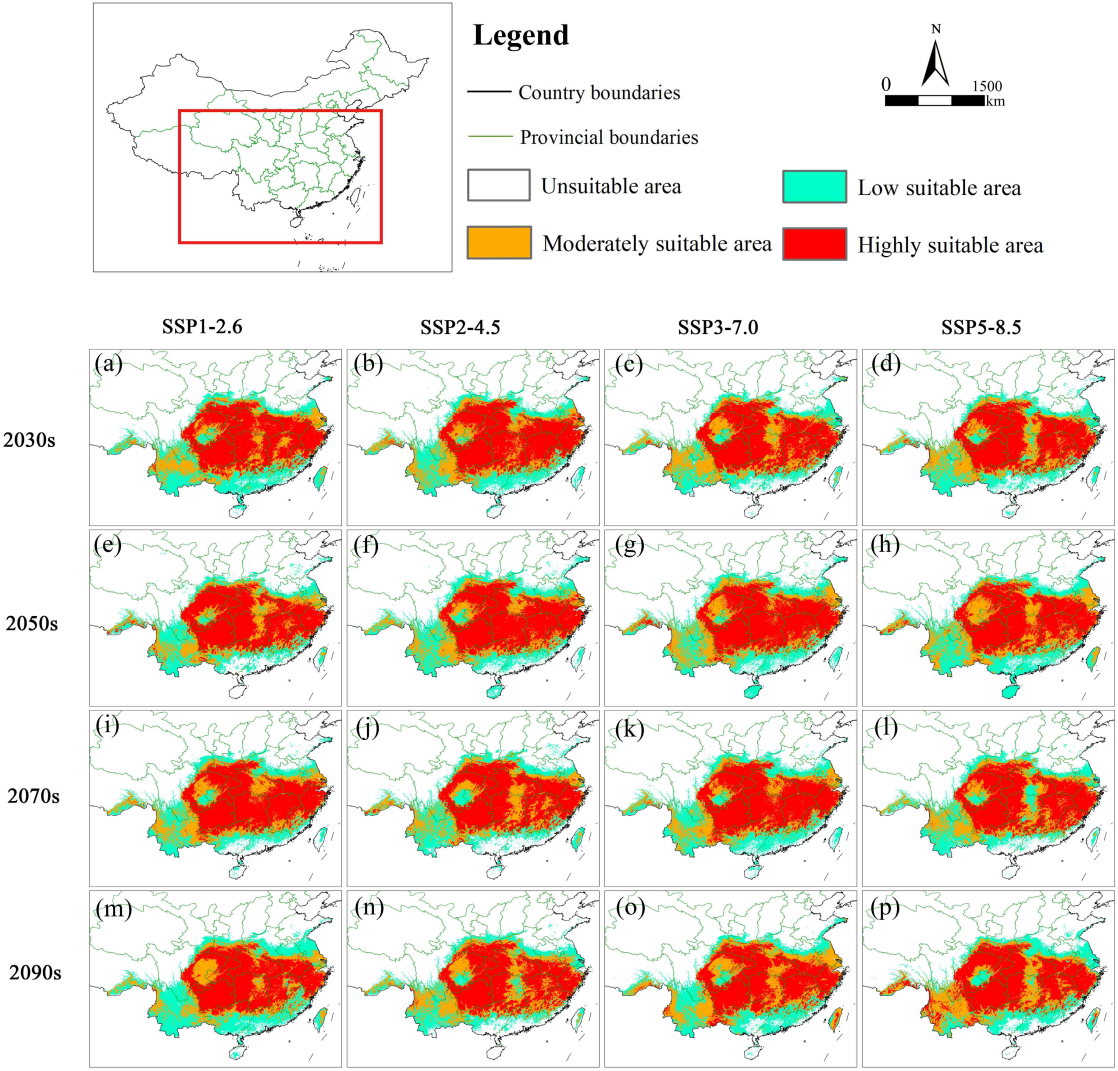
Prediction of changes in the suitable area of *Emmenopterys henryi* in future scenarios by ensemble models. (a) 2030s‐SSP1‐2.6; (b) 2030s‐SSP2‐4.5; (c) 2030s‐SSP3‐7.0; (d) 2030s‐SSP5‐8.5; (e) 2050s‐SSP1‐2.6; (f) 2050s‐SSP2‐4.5; (g) 2050s‐SSP3‐7.0; (h) 2050s‐SSP5‐8.5; (i) 2070s‐SSP1‐2.6; (j) 2070s‐SSP2‐4.5; (k) 2070s‐SSP3‐7.0; (l) 2070s‐SSP5‐8.5; (m) 2090s‐SSP1‐2.6; (n) 2090s‐SSP2‐4.5; (o) 2090s‐SSP3‐7.0; and (p) 2090s‐SSP5‐8.5.

In the future, the suitable area of *E. henryi* was mainly concentrated in eastern China (i.e., southern Jiangsu, southern Anhui, Zhejiang and Jiangxi), central China (i.e., eastern and western Hubei, Hunan), and southwestern China (i.e., eastern Sichuan, central Yunnan, Chongqing and Guizhou) (Figure 6). Under various scenarios, the average suitable area amounted to 169.65 × 10^4^ km^2^, accounting for 17.76% of China's total territory (Table 3).

The suitable area of *E. henryi* would respond similarly under different emission scenarios during the same period. Compared to the current, the 2021–2040 suitable area reduced by 4.27%, (SSP1‐2.6), 2.11% (SSP2‐4.5), 3.46% (SSP3‐7.0), and 6.71% (SSP5‐8.5), respectively. The 2041–2060 suitable area reduced by 7.03% (SSP1‐2.6), 4.11% (SSP2‐4.5), 0.32% (SSP3‐7.0), and 1.24% (SSP5‐8.5), respectively. The 2061–2080 suitable area reduced by 3.08% (SSP1‐2.6), 5.36% (SSP2‐4.5), 3.46% (SSP3‐7.0), and 6.60% (SSP5‐8.5), respectively. And the 2081–2100 suitable area reduced by 7.09% (SSP1‐2.6), 4.38% (SSP2‐4.5), 1.46% (SSP3‐7.0), and 1.62% (SSP5‐8.5), respectively. Collectively, there was a decrease of 15.09% in moderately suitable areas and an increase of 4.21% in highly suitable areas. Hence, the suitable area of *E. henryi* would decrease in all 16 future scenarios.

Except central in China, *E. henryi* populations in eastern and southwestern China responded differently to the future climate scenarios in terms of habitat fragmentation. The habitat fragmentation of *E. henryi* would be reduced in southeastern China (i.e., Jiangsu and Fujian provinces) under the SSP1‐2.6, SSP2‐4.5, and SSP3‐7.0 while it would be aggravated in southwestern China (i.e., Yunnan) under the same conditions. Nevertheless, *E. henryi* populations from the above two parts in China become more fragmented under the SSP5‐8.5. Overall, the habitat fragmentation of *E. henryi* in China would increase under the future scenarios.

### 3.5 Centroid Migration of *E. henryi* Under Different Scenarios

**FIGURE 7:**
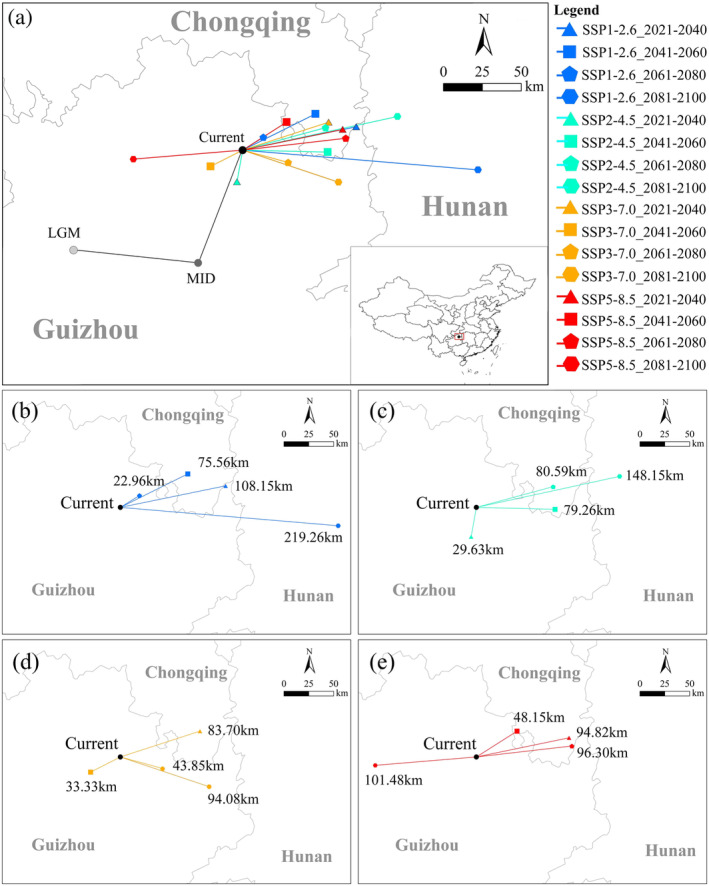
Migration of the core distribution in a suitable area of *Emmenopterys henryi*. (a) Migration of the core distribution in the suitable area of *Emmenopterys henryi* under all climate scenarios. (b) Under SSP1‐2.6; (c) under SSP2‐4.5; (d) under SSP 3‐7.0; and (e) under SSP 5‐8.5.

The current centroid of *E. henryi* population was located in the northern part of Guizhou Province, southwest China (Figure 7a). From the Last Glacial Maximum to the Middle Holocene and then to the current, the centroid migrated southwestward by 79.63 km at first and subsequently northeastward by 77.78 km.

The centroid of this species largely moved northeastward under the nine future climate scenarios (i.e., 2030s, 2050s, 2070s under the SSP1‐2.6; 2070s, 2090s under the SSP2‐4.5; 2030s under the SSP3‐7.0; and 2030s, 2050s, 2070s under the SSP5‐8.5). Among them, the migration distance was the longest in SSP2‐4.5‐2090s scenario, moving 148.15 km to the northeast and reaching the west of Hunan Province. The centroid moved southeastward under the four future climate scenarios (i.e., 2090s under the SSP1‐2.6, 2050s under the SSP2‐4.5; 2070s, 2090s under the SSP3‐7.0). It migrated 219.26 km under the SSP1‐2.6‐2090s scenario, and the centroid was located in the western part of Hunan Province. The centroid moved southwestward under the other three scenarios (i.e., 2030s under the SSP2‐4.5, 2050s under the SSP3‐7.0 and 2090s under the SSP5‐8.5), among which, it migrated the farthest under the SSP5‐8.5‐2090s scenario, migrating 101.48 km to the southeast and reaching northeastern Guizhou Province. Overall, *E. henryi* had a similar tendency to move to the northeast in past and future scenarios.

## 4 Discussion

### 4.1 Model Evaluation and Key Factors

In this study, we employed the Biomod2 and MaxEnt to predict the potential distribution of *E. henryi*, and evaluated the model performance using both AUC and TSS. For one thing, as a tertiary relict tree species (Li 2018), the niches of *E. henryi* remain conserved over time and space. For another thing, for the ensemble model, the final AUC and TSS values were 0.9715 and 0.8405, respectively, while for MaxEnt they were 0.9240 and 0.7897, respectively (Table 1). Both the two models achieve excellent levels; moreover, the outcome provided by Biomod2 is more accurate than MaxEnt. This indicates that an ensemble model (i.e., multi‐model ensembles) may be much better than an individual model for projecting the distribution of *E. henryi*. Besides, the known occurrence records of *E. henryi* (Figure 2) are consistent with the current distribution predicted by the Biomod2 package (Figure 4), which also indicates that the ensemble model is reliable. In addition, it has been contended that ensemble SDMs can produce better prediction outcomes than single model when building alternative models with the same climate variables (Stewart et al. 2022). Our results confirm that an ensemble model formed by several pre‐selected modeling approaches has better predictive performance than other individual model in the case of *E. henryi*, which is consistent with Valavi et al. (2023).

Our results show that the first three key environmental variables influencing the distribution of the *E. henryi* population are the minimum temperature of the coldest month (BIO6), the precipitation of warmest quarter (BIO18), and the temperature seasonality (BIO4), respectively. They are all bioclimatic variables, which indicating that climatic factors may play a more important role in limiting the potential distribution of *E. henryi* than soil or topographical factors. Among the 22 candidate variables, BIO6 has the biggest important value, up to 51.12% (Table 2). This indicates that temperature‐related variables may have much greater effect on the potential distribution of *E. henryi* than precipitation‐related variables. Our model outputs are very similar to the result from Tang et al. (2023), in which BIO9 contributes the most. However, it is quite different from Chen et al. (2022), in which T‐clay contributes the most. A major reason for such a difference can be attributed to sample representativeness. Indeed, only 122 occurrence records were used in MaxEnt modeling by Tang et al. (2023), and only 156 ones, which covered four provinces in southwestern China, were used in MaxEnt by Chen et al. (2022).

According to species‐response curves, *E. henryi* prefers the habitats with a minimum temperature of the coldest month from −4°C to 2°C (Figure 3). This is in line with the experimental results of its seed germination. It is reported that the germination rate of *E. henryi* may slow down and its germination duration increase with the temperature decreasing (Yang 2007). In addition, *E. henryi* will yield few flowers and bear few fruit sets when the temperature is too low, thus affecting its growth and development (Guo, Li, and Lin 2015).

### 4.2 Current Suitable Area of *E. henryi*

According to the results of the ensemble model prediction, the suitable area of *E. henryi* in the current scenario is 176.53 × 10^4^ km^2^, accounting for 18.48% of the national territory. It is higher than the result projected by the MaxEnt model (i.e., 59.91 × 10^4^ km^2^, 6.27% of the national territory). Moreover, our result from the multi‐model ensemble is higher than those derived from individual models (i.e., MaxEnt) (Chen et al. 2022; Tang et al. 2023). Meanwhile, our result is also higher than that reported by Li et al. (2024), which is just 43.89 × 10^4^ km^2^, including the moderately and highly suitable area.

The ensemble model predicted that the current suitable area of *E. henryi* covered 19 provinces in China (i.e., Anhui, Fujian, Jiangsu, Jiangxi, Taiwan, Zhejiang in East China; Guangdong, Guangxi in South China; Shanxi in North China; Henan, Hubei, Hunan in Central China; Gansu, Shaanxi in Northwest China; Chongqing, Guizhou, Sichuan, Tibet, Yunnan in Southwest China) (Figure 4a). Our model outcomes showed that *E. henryi* is likely to occur in Chongqing, Shaanxi, Tibet and Taiwan provinces, besides the 15 provinces recorded by *Flora of China* Vol. 19 (Wu and Raven 2011). For example, a wild *E. henryi* population is reported at Chiyugou, Zhouzhi County, Shaanxi Province (Fan et al. 2012). However, geographical barriers make it difficult for *E. henryi*'s seeds to spread over a long distance from mainland to Taiwan in China. Up to now, no wild population of *E. henryi* has been reported in Taiwan Province (Yang, Liao, and Tang 2008). Therefore, we believe that *E. henryi* is currently distributed in 18 provinces of China, which is mainly concentrated in eastern, central, and southwestern China.

In contrast, the MaxEnt outcomes showed that *E. henryi* presented in 15 provinces of China under the current climate scenario (i.e., Anhui, Fujian, Jiangsu, Jiangxi, Zhejiang in East China; Guangdong, Guangxi in South China; Henan, Hubei, Hunan in Central China; Shaanxi in Northwest China; Chongqing, Guizhou, Sichuan, Yunnan in Southwest China) (Figure 4b). In fact, according to recent field investigation for *E. henryi*, its wild populations also existed at Bifenggou, Wenxian County, Gansu Province, northwestern China (Zhang et al. 2016). Nevertheless, Gansu is not involved in the suitable area forecasted by the MaxEnt. Accordingly, this indicates that the current ensemble modeling is superior to the MaxEnt modeling in terms of predictive results.

We think that there are three main reasons for such a difference between our findings and previous studies. (1) Sampling. Our study was mainly based on an extensive field survey in 2021–2023, with references to data from *Flora of China*, published papers and relevant reports, as well as resource sharing platforms. Firstly, we collected 798 natural occurrence records of *E. henryi* in China. After rarefying, 612 occurrence records of *E. henryi* were obtained for the final modeling. They included 203 occurrence points (taking up 33.17% of 612 occurrence records) in eastern China, 191 ones (31.21%) in southwestern China, 159 ones (25.98%) in central China, 33 ones (5.39%) in southern China, 25 ones (4.08%) in northwestern China; and only one point (0.16%) in northern China (Figure 2). Simultaneously, the sampling records of *E. henryi* were distributed in four of the total seven floristic regions (i.e., subkingdoms) in China (Chen 2019). Therefore, our sampling for *E. henry*i is very representative. In contrast, the existing studies concerning prediction of potential distribution for *E. henryi* have much fewer sampling occurrences than our current study. Accordingly, this may result in under representation in terms of sampling. For example, Li et al. (2024) only used 38 occurrence records of *E. henryi* in an ensemble model. Although their sampling is based on field investigation, it is evident that such data are too few to represent the whole geographical range in China. Specifically, there is only one distribution point in Gansu and Guangxi provinces, two points in Henan, Anhui, Jiangxi, Fujian, Hubei, and Henan provinces, and no occurrence record in Jiangsu Province. More importantly, bias in its sampling would lead to niche truncation of this species. For another example, Chen et al. (2022) employed Maxent to predict suitable areas of *E. henryi* with 156 occurrence records, which were only collected from four provinces in southwestern China. In fact, *E. henryi*, as an endangered tree species endemic to China, mainly occurs in eastern, central, and southwestern China (Wu and Raven 2011). Therefore, we believe that their predicted outcomes hardly reflect the potential geographic distribution of *E. henryi* in China, and furthermore, its response curve may be likely to have the risk of niche truncation.

(2) Data quality. In our analysis, the 612 occurrence records of *E. henryi* are mainly based on the field surveys in recent years as well as the related literature. Therefore, our data sources are extensive and reliable. Moreover, these data cover different regions of China, such as East China, Central China, and Southwest China, so the data used in modeling are highly representative. In addition, we performed ground verification of the model predictions. For example, ground verification was performed for nine sites in East China, including Longtan (Liyang County) in Jiangsu Province; Mountain Huangshan (Huangshan City), Jiuhua Mountain (Qingyang County), and Tiantangzhai (Jinzhai County) in Anhui Province; Tianmu Mountain (Lin'an County), Longwang Mountain (Anji County), and Dapanshan Mountain (Pan'an County) in Zhejiang Province; Taohongling (Pengze County) and Sanqingshan Mountain (Yushan County) in Jiangxi Province. As a result, all these ensure high quality data in this study. In contrast, there are poor‐quality data in other studies on *E. henryi* potential distribution prediction compared with ours. For example, Chen et al. (2022) only collected *E. henryi*'s occurrence records from the southwestern region of China, thereby resulting in sampling bias in China.

(3) Model algorithms. In general, projecting results from ensemble models are better than from individual models (Liu, Graeme, and Matt 2019). The predicted results in our study verify this view. Namely, ensemble forecasts derived by the Biomod2 package are superior to MaxEnt forecasts. Secondly, the MaxEnt in our study is optimized by setting the optimal RM, FC, which improves the accuracy and reliability of model prediction. On the contrary, existing studies like Chen et al. (2022), employed default settings for MaxEnt modeling, which may affect the reliability of the predicted results.

### 4.3 Past and Future Distribution Shift of *E. henryi*

The ensemble model has shown under each of the two past and 16 future climate scenarios the suitable area of *E. henryi* will decrease, ranging from 0.32% (2050s SSP3‐7.0) to 7.09% (2090s SSP1‐2.6). Collectively, its suitable areas will averagely decline by 3.90% in the future conditions (Table 3). Furthermore, *E. henryi* will largely experience increased habitat fragmentation in geographical distribution, especially in Yunnan and Sichuan provinces, southwestern China (Figure 6). Therefore, we think that future climate change may have an adverse effect on *E. henryi* in distribution.

Research has shown that most tree species may shift latitude or elevation range in the context of global warming during the Quaternary (Davis and Shaw 2001). The majority of woody species in eastern China presents a tendency for migrating to north in the last three decades (Song et al. 2016). Our results indicate that *E. henryi* will mainly migrate to the northeast in terms of its future suitable areas (Figure 7), which is consistent with the migration directions of these endangered tree species such as *Ammopiptanthus mongolicus* (Du et al. 2021), *Carpinus tientaiensis* (Zhao et al. 2020), *Taxus wallichiana* var. *mairei* and *T. wallichiana* var. *chinensis* (Wu et al. 2022). This may be related to the tree trait that *E. henryi* prefers cool and humid habitats (Zhang and Yi 2023) because the Chinese humid zone is usually situated in the northeastern and southeastern regions of this country (Jiang, Jiang, and Lin 2017).

### 4.4 Implications for Its Conservation and Management

Our findings indicate that the suitable area of *E. henryi* in China is 176.53 × 10^4^ km^2^, mainly distributed in East China (six provinces), Central China (three provinces), and Southwest China (five provinces). Combined *Flora of China* and plant resources survey with our results, we suggest carrying out its wild population survey in Shaanxi, Chongqing, Jiangsu, and other provinces where *E. henryi* has not been recorded.

This tree often blooms only when it grows 20 years, and its advanced individuals produce flowers every 2–4 years, with a low seed setting rate. This species has small winged seeds in its capsules, and very low seed germination rate in the stand. Therefore, all these may result in its low recruitment in the wild. Niu et al. (2021) applied SSR molecular markers to study the genetic diversity with six population samples, which all come from only one province, and contended that these populations had high genetic diversity. Zhang et al. (2016) used AFLPs and ITS molecular markers to reveal its genetic diversity with 38 population samples from 14 provinces, and concluded that this tree had high genetic diversity at the species level but had low genetic diversity at the population level. Based on the predictive suitable area of *E. henryi*, we think that the latter result is much more credible than the first concerning its genetic diversity.

Our findings also indicate that under the future climate scenario, the suitable area of *E. henryi* is expected to decrease, showing a trend of migration to the northeast in China. Therefore, as a national secondary key protection of wild plant, it is necessary to take into account the adverse effect of climate change on its distribution in conjunction with its tree traits and habitat requirements when carrying out *ex situ* protection, introduction, and cultivation in the future. For example, it is inappropriate to choose the cultivation site in the southern margin of the current distribution area.

## 5 Conclusion

In view of the major shortcomings of current research (i.e., sampling representation, single algorithm), we first collected 612 occurrence records (including 17 provinces) of endangered *E. henryi* according to field survey and consulting literature and related 22 environmental variables, we then applied the forecasting platform Biomod2 comprising nine individual models to predict its current and future potential distribution in China. The ensemble model results showed that the main environmental variables affecting this species were Min temperature of coldest month (BIO6), indicating that temperature‐related variables may have much greater effect on the potential distribution of *E. henryi* than precipitation, topography, and soil‐related variables. The current potential distribution area from ensemble models is 176.53 × 10^4^ km^2^, which is larger than from MaxEnt, and it is mainly concentrated in eastern, central, and southwestern China. Moreover, climate change may have an adverse effect on its potential distribution. Therefore, we suggest conducting a supplementary investigation of its wild population in Shaanxi, Chongqing, Jiangsu, and other provinces where *E. henryi* has not been recorded but within the forecasted suitable area. In addition, for endangered species with large distribution area, we should consider employing an ensemble model formed by multiple models as much as possible, so as to improve the reliability and accuracy of species distribution prediction.

## Author Contributions

**Hanwei Cai:** data curation (equal), formal analysis (equal), writing – original draft (equal). **Guangfu Zhang:** conceptualization (lead), investigation (lead), writing – review and editing (lead).

## Conflicts of Interest

The authors declare no conflicts of interest.

## Supporting information


Appendix S1.

## Data Availability

The original data are contained within the article and appendix.
