# Supplementary material for: Predicting the Potential Distribution of Rare and Endangered Emmenopterys henryi in China Under Climate Change
Source: Ecol Evol. 2024 Oct 13;14(10):e70403. doi: 10.1002/ece3.70403 (PMC11471803; doi:10.1002/ece3.70403)
Supplement: Supplementary file 1 — Appendix S1. [file ECE3-14-e70403-s001.docx]

**Appendix**

**Table S1 Detailed distribution records of *Emmenopterys henryi* in this study.**

| **Species** | **Longitude (°)** | **Latitude (°)** |
| --- | --- | --- |
| *Emmenopterys henryi* | 97.855 | 24.012 |
| *Emmenopterys henryi* | 98.960 | 25.750 |
| *Emmenopterys henryi* | 99.740 | 24.026 |
| *Emmenopterys henryi* | 100.354 | 24.537 |
| *Emmenopterys henryi* | 100.385 | 21.843 |
| *Emmenopterys henryi* | 100.799 | 26.482 |
| *Emmenopterys henryi* | 101.309 | 27.635 |
| *Emmenopterys henryi* | 101.402 | 25.016 |
| *Emmenopterys henryi* | 101.500 | 29.000 |
| *Emmenopterys henryi* | 101.855 | 26.919 |
| *Emmenopterys henryi* | 101.953 | 29.994 |
| *Emmenopterys henryi* | 102.272 | 29.875 |
| *Emmenopterys henryi* | 102.350 | 24.266 |
| *Emmenopterys henryi* | 102.353 | 28.893 |
| *Emmenopterys henryi* | 102.366 | 29.900 |
| *Emmenopterys henryi* | 102.376 | 29.038 |
| *Emmenopterys henryi* | 102.389 | 24.330 |
| *Emmenopterys henryi* | 102.433 | 25.233 |
| *Emmenopterys henryi* | 102.450 | 30.167 |
| *Emmenopterys henryi* | 102.456 | 24.996 |
| *Emmenopterys henryi* | 102.480 | 24.930 |
| *Emmenopterys henryi* | 102.500 | 28.630 |
| *Emmenopterys henryi* | 102.555 | 30.119 |
| *Emmenopterys henryi* | 102.597 | 30.417 |
| *Emmenopterys henryi* | 102.659 | 30.420 |
| *Emmenopterys henryi* | 102.686 | 30.576 |
| *Emmenopterys henryi* | 102.750 | 30.060 |
| *Emmenopterys henryi* | 102.776 | 28.981 |
| *Emmenopterys henryi* | 102.810 | 30.360 |
| *Emmenopterys henryi* | 102.912 | 30.537 |
| *Emmenopterys henryi* | 102.939 | 24.080 |
| *Emmenopterys henryi* | 103.066 | 25.650 |
| *Emmenopterys henryi* | 103.234 | 23.863 |
| *Emmenopterys henryi* | 103.300 | 29.500 |
| *Emmenopterys henryi* | 103.357 | 29.547 |
| *Emmenopterys henryi* | 103.390 | 29.490 |
| *Emmenopterys henryi* | 103.425 | 29.554 |
| *Emmenopterys henryi* | 103.508 | 29.335 |
| *Emmenopterys henryi* | 103.520 | 29.594 |
| *Emmenopterys henryi* | 103.528 | 30.200 |
| *Emmenopterys henryi* | 103.557 | 30.910 |
| *Emmenopterys henryi* | 103.630 | 31.003 |
| *Emmenopterys henryi* | 103.670 | 28.680 |
| *Emmenopterys henryi* | 103.699 | 32.055 |
| *Emmenopterys henryi* | 103.726 | 32.453 |
| *Emmenopterys henryi* | 103.815 | 29.901 |
| *Emmenopterys henryi* | 103.828 | 30.053 |
| *Emmenopterys henryi* | 103.834 | 32.727 |
| *Emmenopterys henryi* | 103.959 | 23.377 |
| *Emmenopterys henryi* | 104.080 | 30.710 |
| *Emmenopterys henryi* | 104.107 | 32.176 |
| *Emmenopterys henryi* | 104.266 | 31.816 |
| *Emmenopterys henryi* | 104.322 | 32.113 |
| *Emmenopterys henryi* | 104.545 | 27.383 |
| *Emmenopterys henryi* | 104.550 | 32.440 |
| *Emmenopterys henryi* | 104.580 | 28.110 |
| *Emmenopterys henryi* | 104.667 | 28.006 |
| *Emmenopterys henryi* | 104.715 | 27.919 |
| *Emmenopterys henryi* | 104.724 | 27.126 |
| *Emmenopterys henryi* | 104.750 | 27.466 |
| *Emmenopterys henryi* | 104.769 | 27.900 |
| *Emmenopterys henryi* | 104.782 | 23.383 |
| *Emmenopterys henryi* | 104.783 | 23.183 |
| *Emmenopterys henryi* | 104.843 | 25.253 |
| *Emmenopterys henryi* | 104.869 | 24.669 |
| *Emmenopterys henryi* | 104.870 | 27.440 |
| *Emmenopterys henryi* | 104.885 | 27.325 |
| *Emmenopterys henryi* | 104.890 | 25.090 |
| *Emmenopterys henryi* | 104.913 | 26.020 |
| *Emmenopterys henryi* | 104.952 | 24.618 |
| *Emmenopterys henryi* | 105.147 | 32.743 |
| *Emmenopterys henryi* | 105.163 | 32.655 |
| *Emmenopterys henryi* | 105.266 | 33.067 |
| *Emmenopterys henryi* | 105.330 | 24.680 |
| *Emmenopterys henryi* | 105.359 | 27.664 |
| *Emmenopterys henryi* | 105.367 | 27.382 |
| *Emmenopterys henryi* | 105.415 | 27.593 |
| *Emmenopterys henryi* | 105.439 | 32.753 |
| *Emmenopterys henryi* | 105.450 | 33.204 |
| *Emmenopterys henryi* | 105.541 | 23.520 |
| *Emmenopterys henryi* | 105.587 | 24.576 |
| *Emmenopterys henryi* | 105.593 | 23.269 |
| *Emmenopterys henryi* | 105.604 | 24.981 |
| *Emmenopterys henryi* | 105.624 | 33.452 |
| *Emmenopterys henryi* | 105.662 | 24.986 |
| *Emmenopterys henryi* | 105.694 | 27.617 |
| *Emmenopterys henryi* | 105.701 | 32.957 |
| *Emmenopterys henryi* | 105.756 | 27.388 |
| *Emmenopterys henryi* | 105.830 | 23.400 |
| *Emmenopterys henryi* | 105.836 | 25.331 |
| *Emmenopterys henryi* | 105.848 | 26.169 |
| *Emmenopterys henryi* | 105.886 | 26.101 |
| *Emmenopterys henryi* | 105.911 | 30.680 |
| *Emmenopterys henryi* | 106.176 | 26.568 |
| *Emmenopterys henryi* | 106.217 | 25.147 |
| *Emmenopterys henryi* | 106.250 | 26.410 |
| *Emmenopterys henryi* | 106.309 | 25.482 |
| *Emmenopterys henryi* | 106.330 | 26.670 |
| *Emmenopterys henryi* | 106.350 | 29.794 |
| *Emmenopterys henryi* | 106.354 | 26.816 |
| *Emmenopterys henryi* | 106.401 | 27.736 |
| *Emmenopterys henryi* | 106.414 | 33.241 |
| *Emmenopterys henryi* | 106.422 | 29.819 |
| *Emmenopterys henryi* | 106.431 | 26.543 |
| *Emmenopterys henryi* | 106.450 | 33.130 |
| *Emmenopterys henryi* | 106.482 | 33.063 |
| *Emmenopterys henryi* | 106.488 | 33.318 |
| *Emmenopterys henryi* | 106.520 | 26.232 |
| *Emmenopterys henryi* | 106.528 | 28.452 |
| *Emmenopterys henryi* | 106.537 | 24.931 |
| *Emmenopterys henryi* | 106.550 | 24.780 |
| *Emmenopterys henryi* | 106.612 | 26.450 |
| *Emmenopterys henryi* | 106.615 | 24.607 |
| *Emmenopterys henryi* | 106.623 | 27.115 |
| *Emmenopterys henryi* | 106.659 | 27.018 |
| *Emmenopterys henryi* | 106.667 | 27.942 |
| *Emmenopterys henryi* | 106.676 | 26.508 |
| *Emmenopterys henryi* | 106.678 | 26.440 |
| *Emmenopterys henryi* | 106.687 | 32.927 |
| *Emmenopterys henryi* | 106.694 | 27.104 |
| *Emmenopterys henryi* | 106.716 | 26.835 |
| *Emmenopterys henryi* | 106.754 | 26.633 |
| *Emmenopterys henryi* | 106.757 | 24.118 |
| *Emmenopterys henryi* | 106.815 | 28.433 |
| *Emmenopterys henryi* | 106.821 | 28.141 |
| *Emmenopterys henryi* | 106.832 | 26.192 |
| *Emmenopterys henryi* | 106.840 | 22.715 |
| *Emmenopterys henryi* | 106.864 | 29.025 |
| *Emmenopterys henryi* | 107.070 | 25.156 |
| *Emmenopterys henryi* | 107.080 | 29.021 |
| *Emmenopterys henryi* | 107.085 | 29.070 |
| *Emmenopterys henryi* | 107.090 | 29.150 |
| *Emmenopterys henryi* | 107.102 | 26.766 |
| *Emmenopterys henryi* | 107.110 | 22.760 |
| *Emmenopterys henryi* | 107.117 | 28.840 |
| *Emmenopterys henryi* | 107.133 | 28.410 |
| *Emmenopterys henryi* | 107.161 | 28.224 |
| *Emmenopterys henryi* | 107.180 | 27.950 |
| *Emmenopterys henryi* | 107.187 | 29.015 |
| *Emmenopterys henryi* | 107.198 | 22.683 |
| *Emmenopterys henryi* | 107.213 | 29.263 |
| *Emmenopterys henryi* | 107.226 | 32.845 |
| *Emmenopterys henryi* | 107.282 | 28.588 |
| *Emmenopterys henryi* | 107.352 | 25.694 |
| *Emmenopterys henryi* | 107.383 | 28.568 |
| *Emmenopterys henryi* | 107.386 | 27.936 |
| *Emmenopterys henryi* | 107.389 | 27.496 |
| *Emmenopterys henryi* | 107.405 | 28.797 |
| *Emmenopterys henryi* | 107.416 | 33.733 |
| *Emmenopterys henryi* | 107.438 | 22.976 |
| *Emmenopterys henryi* | 107.450 | 32.033 |
| *Emmenopterys henryi* | 107.460 | 28.539 |
| *Emmenopterys henryi* | 107.471 | 27.069 |
| *Emmenopterys henryi* | 107.472 | 25.618 |
| *Emmenopterys henryi* | 107.530 | 25.820 |
| *Emmenopterys henryi* | 107.572 | 29.114 |
| *Emmenopterys henryi* | 107.582 | 28.221 |
| *Emmenopterys henryi* | 107.592 | 28.511 |
| *Emmenopterys henryi* | 107.639 | 26.980 |
| *Emmenopterys henryi* | 107.659 | 25.995 |
| *Emmenopterys henryi* | 107.710 | 27.950 |
| *Emmenopterys henryi* | 107.858 | 34.134 |
| *Emmenopterys henryi* | 107.878 | 25.150 |
| *Emmenopterys henryi* | 107.880 | 25.410 |
| *Emmenopterys henryi* | 107.921 | 28.060 |
| *Emmenopterys henryi* | 107.925 | 25.306 |
| *Emmenopterys henryi* | 108.000 | 27.300 |
| *Emmenopterys henryi* | 108.056 | 25.260 |
| *Emmenopterys henryi* | 108.056 | 27.533 |
| *Emmenopterys henryi* | 108.060 | 27.344 |
| *Emmenopterys henryi* | 108.078 | 26.105 |
| *Emmenopterys henryi* | 108.110 | 28.260 |
| *Emmenopterys henryi* | 108.177 | 28.212 |
| *Emmenopterys henryi* | 108.203 | 26.390 |
| *Emmenopterys henryi* | 108.220 | 27.520 |
| *Emmenopterys henryi* | 108.292 | 27.646 |
| *Emmenopterys henryi* | 108.329 | 33.970 |
| *Emmenopterys henryi* | 108.340 | 32.263 |
| *Emmenopterys henryi* | 108.349 | 34.014 |
| *Emmenopterys henryi* | 108.400 | 27.990 |
| *Emmenopterys henryi* | 108.401 | 34.184 |
| *Emmenopterys henryi* | 108.404 | 26.498 |
| *Emmenopterys henryi* | 108.439 | 32.500 |
| *Emmenopterys henryi* | 108.442 | 32.293 |
| *Emmenopterys henryi* | 108.444 | 27.751 |
| *Emmenopterys henryi* | 108.462 | 27.887 |
| *Emmenopterys henryi* | 108.480 | 27.007 |
| *Emmenopterys henryi* | 108.483 | 27.466 |
| *Emmenopterys henryi* | 108.490 | 28.550 |
| *Emmenopterys henryi* | 108.490 | 32.650 |
| *Emmenopterys henryi* | 108.507 | 26.603 |
| *Emmenopterys henryi* | 108.514 | 28.087 |
| *Emmenopterys henryi* | 108.516 | 27.898 |
| *Emmenopterys henryi* | 108.530 | 32.510 |
| *Emmenopterys henryi* | 108.533 | 27.518 |
| *Emmenopterys henryi* | 108.600 | 27.933 |
| *Emmenopterys henryi* | 108.658 | 28.031 |
| *Emmenopterys henryi* | 108.680 | 30.436 |
| *Emmenopterys henryi* | 108.689 | 28.087 |
| *Emmenopterys henryi* | 108.692 | 27.917 |
| *Emmenopterys henryi* | 108.732 | 30.024 |
| *Emmenopterys henryi* | 108.736 | 26.396 |
| *Emmenopterys henryi* | 108.737 | 26.471 |
| *Emmenopterys henryi* | 108.748 | 29.913 |
| *Emmenopterys henryi* | 108.814 | 27.075 |
| *Emmenopterys henryi* | 108.888 | 28.020 |
| *Emmenopterys henryi* | 108.930 | 30.290 |
| *Emmenopterys henryi* | 108.937 | 27.681 |
| *Emmenopterys henryi* | 109.047 | 30.023 |
| *Emmenopterys henryi* | 109.089 | 27.801 |
| *Emmenopterys henryi* | 109.128 | 30.034 |
| *Emmenopterys henryi* | 109.135 | 28.253 |
| *Emmenopterys henryi* | 109.154 | 26.755 |
| *Emmenopterys henryi* | 109.195 | 26.679 |
| *Emmenopterys henryi* | 109.240 | 26.657 |
| *Emmenopterys henryi* | 109.286 | 28.982 |
| *Emmenopterys henryi* | 109.319 | 29.716 |
| *Emmenopterys henryi* | 109.404 | 28.720 |
| *Emmenopterys henryi* | 109.407 | 27.248 |
| *Emmenopterys henryi* | 109.419 | 29.725 |
| *Emmenopterys henryi* | 109.432 | 31.916 |
| *Emmenopterys henryi* | 109.446 | 30.659 |
| *Emmenopterys henryi* | 109.452 | 27.509 |
| *Emmenopterys henryi* | 109.465 | 31.017 |
| *Emmenopterys henryi* | 109.470 | 30.290 |
| *Emmenopterys henryi* | 109.480 | 29.990 |
| *Emmenopterys henryi* | 109.488 | 30.447 |
| *Emmenopterys henryi* | 109.498 | 28.111 |
| *Emmenopterys henryi* | 109.535 | 29.706 |
| *Emmenopterys henryi* | 109.541 | 32.086 |
| *Emmenopterys henryi* | 109.582 | 28.347 |
| *Emmenopterys henryi* | 109.590 | 27.950 |
| *Emmenopterys henryi* | 109.649 | 27.606 |
| *Emmenopterys henryi* | 109.702 | 29.644 |
| *Emmenopterys henryi* | 109.705 | 29.858 |
| *Emmenopterys henryi* | 109.715 | 29.990 |
| *Emmenopterys henryi* | 109.718 | 29.695 |
| *Emmenopterys henryi* | 109.728 | 29.008 |
| *Emmenopterys henryi* | 109.729 | 30.038 |
| *Emmenopterys henryi* | 109.730 | 29.324 |
| *Emmenopterys henryi* | 109.756 | 29.907 |
| *Emmenopterys henryi* | 109.776 | 29.086 |
| *Emmenopterys henryi* | 109.798 | 25.496 |
| *Emmenopterys henryi* | 109.809 | 29.136 |
| *Emmenopterys henryi* | 109.818 | 30.142 |
| *Emmenopterys henryi* | 109.818 | 28.817 |
| *Emmenopterys henryi* | 109.840 | 29.000 |
| *Emmenopterys henryi* | 109.843 | 29.881 |
| *Emmenopterys henryi* | 109.859 | 31.939 |
| *Emmenopterys henryi* | 109.863 | 25.731 |
| *Emmenopterys henryi* | 109.868 | 30.039 |
| *Emmenopterys henryi* | 109.883 | 25.822 |
| *Emmenopterys henryi* | 109.886 | 25.651 |
| *Emmenopterys henryi* | 110.025 | 29.673 |
| *Emmenopterys henryi* | 110.030 | 29.880 |
| *Emmenopterys henryi* | 110.040 | 25.740 |
| *Emmenopterys henryi* | 110.050 | 29.750 |
| *Emmenopterys henryi* | 110.068 | 28.665 |
| *Emmenopterys henryi* | 110.071 | 31.283 |
| *Emmenopterys henryi* | 110.083 | 31.451 |
| *Emmenopterys henryi* | 110.087 | 31.206 |
| *Emmenopterys henryi* | 110.089 | 28.761 |
| *Emmenopterys henryi* | 110.118 | 28.817 |
| *Emmenopterys henryi* | 110.150 | 29.400 |
| *Emmenopterys henryi* | 110.154 | 28.908 |
| *Emmenopterys henryi* | 110.190 | 25.240 |
| *Emmenopterys henryi* | 110.201 | 30.088 |
| *Emmenopterys henryi* | 110.216 | 31.716 |
| *Emmenopterys henryi* | 110.224 | 30.019 |
| *Emmenopterys henryi* | 110.224 | 26.363 |
| *Emmenopterys henryi* | 110.233 | 30.669 |
| *Emmenopterys henryi* | 110.241 | 33.011 |
| *Emmenopterys henryi* | 110.257 | 28.808 |
| *Emmenopterys henryi* | 110.285 | 27.336 |
| *Emmenopterys henryi* | 110.308 | 26.362 |
| *Emmenopterys henryi* | 110.315 | 24.945 |
| *Emmenopterys henryi* | 110.316 | 27.166 |
| *Emmenopterys henryi* | 110.324 | 30.718 |
| *Emmenopterys henryi* | 110.340 | 31.040 |
| *Emmenopterys henryi* | 110.352 | 26.844 |
| *Emmenopterys henryi* | 110.365 | 25.589 |
| *Emmenopterys henryi* | 110.371 | 31.435 |
| *Emmenopterys henryi* | 110.373 | 25.861 |
| *Emmenopterys henryi* | 110.374 | 30.817 |
| *Emmenopterys henryi* | 110.400 | 31.016 |
| *Emmenopterys henryi* | 110.406 | 27.310 |
| *Emmenopterys henryi* | 110.416 | 29.316 |
| *Emmenopterys henryi* | 110.441 | 31.460 |
| *Emmenopterys henryi* | 110.449 | 30.420 |
| *Emmenopterys henryi* | 110.465 | 24.409 |
| *Emmenopterys henryi* | 110.500 | 31.500 |
| *Emmenopterys henryi* | 110.510 | 28.323 |
| *Emmenopterys henryi* | 110.512 | 28.998 |
| *Emmenopterys henryi* | 110.520 | 24.691 |
| *Emmenopterys henryi* | 110.529 | 31.317 |
| *Emmenopterys henryi* | 110.530 | 29.373 |
| *Emmenopterys henryi* | 110.567 | 28.325 |
| *Emmenopterys henryi* | 110.570 | 27.067 |
| *Emmenopterys henryi* | 110.571 | 29.920 |
| *Emmenopterys henryi* | 110.580 | 25.600 |
| *Emmenopterys henryi* | 110.586 | 26.477 |
| *Emmenopterys henryi* | 110.593 | 30.200 |
| *Emmenopterys henryi* | 110.601 | 31.650 |
| *Emmenopterys henryi* | 110.607 | 28.404 |
| *Emmenopterys henryi* | 110.611 | 25.680 |
| *Emmenopterys henryi* | 110.621 | 26.644 |
| *Emmenopterys henryi* | 110.628 | 31.371 |
| *Emmenopterys henryi* | 110.639 | 26.199 |
| *Emmenopterys henryi* | 110.640 | 26.728 |
| *Emmenopterys henryi* | 110.670 | 31.740 |
| *Emmenopterys henryi* | 110.676 | 31.606 |
| *Emmenopterys henryi* | 110.689 | 29.997 |
| *Emmenopterys henryi* | 110.714 | 29.323 |
| *Emmenopterys henryi* | 110.729 | 25.336 |
| *Emmenopterys henryi* | 110.779 | 29.430 |
| *Emmenopterys henryi* | 110.780 | 31.330 |
| *Emmenopterys henryi* | 110.781 | 30.843 |
| *Emmenopterys henryi* | 110.783 | 26.291 |
| *Emmenopterys henryi* | 110.833 | 29.034 |
| *Emmenopterys henryi* | 110.840 | 26.430 |
| *Emmenopterys henryi* | 110.870 | 30.220 |
| *Emmenopterys henryi* | 110.882 | 31.053 |
| *Emmenopterys henryi* | 110.883 | 31.214 |
| *Emmenopterys henryi* | 110.888 | 31.596 |
| *Emmenopterys henryi* | 110.966 | 26.416 |
| *Emmenopterys henryi* | 110.970 | 30.820 |
| *Emmenopterys henryi* | 110.990 | 32.400 |
| *Emmenopterys henryi* | 111.017 | 33.238 |
| *Emmenopterys henryi* | 111.025 | 26.534 |
| *Emmenopterys henryi* | 111.069 | 32.467 |
| *Emmenopterys henryi* | 111.092 | 26.390 |
| *Emmenopterys henryi* | 111.120 | 29.420 |
| *Emmenopterys henryi* | 111.123 | 28.602 |
| *Emmenopterys henryi* | 111.281 | 30.694 |
| *Emmenopterys henryi* | 111.284 | 30.171 |
| *Emmenopterys henryi* | 111.370 | 29.590 |
| *Emmenopterys henryi* | 111.371 | 33.465 |
| *Emmenopterys henryi* | 111.441 | 33.483 |
| *Emmenopterys henryi* | 111.587 | 31.594 |
| *Emmenopterys henryi* | 111.600 | 33.600 |
| *Emmenopterys henryi* | 111.624 | 33.673 |
| *Emmenopterys henryi* | 111.643 | 33.720 |
| *Emmenopterys henryi* | 111.741 | 32.414 |
| *Emmenopterys henryi* | 111.755 | 27.524 |
| *Emmenopterys henryi* | 111.766 | 33.719 |
| *Emmenopterys henryi* | 111.791 | 33.375 |
| *Emmenopterys henryi* | 111.864 | 33.744 |
| *Emmenopterys henryi* | 111.885 | 33.454 |
| *Emmenopterys henryi* | 111.933 | 34.037 |
| *Emmenopterys henryi* | 111.936 | 33.517 |
| *Emmenopterys henryi* | 111.944 | 33.634 |
| *Emmenopterys henryi* | 111.987 | 33.476 |
| *Emmenopterys henryi* | 111.991 | 33.647 |
| *Emmenopterys henryi* | 111.998 | 33.704 |
| *Emmenopterys henryi* | 112.106 | 33.569 |
| *Emmenopterys henryi* | 112.148 | 27.267 |
| *Emmenopterys henryi* | 112.243 | 33.720 |
| *Emmenopterys henryi* | 112.313 | 34.056 |
| *Emmenopterys henryi* | 112.361 | 34.671 |
| *Emmenopterys henryi* | 112.433 | 25.129 |
| *Emmenopterys henryi* | 112.600 | 27.300 |
| *Emmenopterys henryi* | 112.675 | 27.301 |
| *Emmenopterys henryi* | 112.729 | 27.246 |
| *Emmenopterys henryi* | 113.042 | 32.459 |
| *Emmenopterys henryi* | 113.080 | 31.313 |
| *Emmenopterys henryi* | 113.160 | 30.660 |
| *Emmenopterys henryi* | 113.269 | 32.397 |
| *Emmenopterys henryi* | 113.378 | 35.412 |
| *Emmenopterys henryi* | 113.400 | 32.360 |
| *Emmenopterys henryi* | 113.595 | 32.352 |
| *Emmenopterys henryi* | 113.609 | 31.579 |
| *Emmenopterys henryi* | 113.629 | 33.013 |
| *Emmenopterys henryi* | 113.731 | 26.008 |
| *Emmenopterys henryi* | 113.818 | 30.756 |
| *Emmenopterys henryi* | 113.884 | 29.562 |
| *Emmenopterys henryi* | 113.938 | 31.827 |
| *Emmenopterys henryi* | 113.948 | 32.104 |
| *Emmenopterys henryi* | 113.961 | 29.065 |
| *Emmenopterys henryi* | 114.016 | 26.558 |
| *Emmenopterys henryi* | 114.040 | 29.530 |
| *Emmenopterys henryi* | 114.056 | 31.822 |
| *Emmenopterys henryi* | 114.068 | 29.278 |
| *Emmenopterys henryi* | 114.072 | 28.426 |
| *Emmenopterys henryi* | 114.079 | 25.959 |
| *Emmenopterys henryi* | 114.109 | 31.847 |
| *Emmenopterys henryi* | 114.116 | 28.391 |
| *Emmenopterys henryi* | 114.146 | 26.019 |
| *Emmenopterys henryi* | 114.151 | 29.222 |
| *Emmenopterys henryi* | 114.183 | 26.616 |
| *Emmenopterys henryi* | 114.194 | 26.800 |
| *Emmenopterys henryi* | 114.196 | 26.368 |
| *Emmenopterys henryi* | 114.200 | 28.450 |
| *Emmenopterys henryi* | 114.200 | 28.533 |
| *Emmenopterys henryi* | 114.202 | 31.905 |
| *Emmenopterys henryi* | 114.264 | 28.485 |
| *Emmenopterys henryi* | 114.283 | 26.933 |
| *Emmenopterys henryi* | 114.304 | 29.117 |
| *Emmenopterys henryi* | 114.350 | 28.750 |
| *Emmenopterys henryi* | 114.354 | 30.534 |
| *Emmenopterys henryi* | 114.370 | 28.520 |
| *Emmenopterys henryi* | 114.410 | 29.010 |
| *Emmenopterys henryi* | 114.416 | 25.880 |
| *Emmenopterys henryi* | 114.435 | 26.218 |
| *Emmenopterys henryi* | 114.457 | 26.016 |
| *Emmenopterys henryi* | 114.469 | 28.520 |
| *Emmenopterys henryi* | 114.490 | 29.600 |
| *Emmenopterys henryi* | 114.510 | 26.320 |
| *Emmenopterys henryi* | 114.545 | 28.502 |
| *Emmenopterys henryi* | 114.560 | 29.030 |
| *Emmenopterys henryi* | 114.563 | 29.364 |
| *Emmenopterys henryi* | 114.591 | 26.380 |
| *Emmenopterys henryi* | 114.601 | 31.542 |
| *Emmenopterys henryi* | 114.645 | 31.603 |
| *Emmenopterys henryi* | 114.651 | 27.171 |
| *Emmenopterys henryi* | 114.661 | 29.396 |
| *Emmenopterys henryi* | 114.680 | 28.916 |
| *Emmenopterys henryi* | 114.681 | 29.518 |
| *Emmenopterys henryi* | 114.721 | 28.802 |
| *Emmenopterys henryi* | 114.733 | 28.891 |
| *Emmenopterys henryi* | 114.741 | 28.715 |
| *Emmenopterys henryi* | 114.766 | 31.581 |
| *Emmenopterys henryi* | 114.780 | 28.380 |
| *Emmenopterys henryi* | 114.786 | 31.654 |
| *Emmenopterys henryi* | 114.787 | 28.843 |
| *Emmenopterys henryi* | 114.793 | 27.479 |
| *Emmenopterys henryi* | 114.865 | 28.843 |
| *Emmenopterys henryi* | 114.875 | 31.617 |
| *Emmenopterys henryi* | 114.955 | 28.957 |
| *Emmenopterys henryi* | 114.981 | 29.094 |
| *Emmenopterys henryi* | 114.989 | 29.542 |
| *Emmenopterys henryi* | 115.010 | 29.380 |
| *Emmenopterys henryi* | 115.058 | 30.775 |
| *Emmenopterys henryi* | 115.090 | 29.260 |
| *Emmenopterys henryi* | 115.313 | 30.947 |
| *Emmenopterys henryi* | 115.329 | 31.400 |
| *Emmenopterys henryi* | 115.359 | 24.815 |
| *Emmenopterys henryi* | 115.364 | 31.619 |
| *Emmenopterys henryi* | 115.387 | 31.474 |
| *Emmenopterys henryi* | 115.390 | 30.780 |
| *Emmenopterys henryi* | 115.486 | 31.625 |
| *Emmenopterys henryi* | 115.490 | 32.010 |
| *Emmenopterys henryi* | 115.491 | 29.054 |
| *Emmenopterys henryi* | 115.524 | 29.189 |
| *Emmenopterys henryi* | 115.546 | 28.055 |
| *Emmenopterys henryi* | 115.566 | 29.558 |
| *Emmenopterys henryi* | 115.587 | 25.043 |
| *Emmenopterys henryi* | 115.589 | 31.690 |
| *Emmenopterys henryi* | 115.589 | 29.087 |
| *Emmenopterys henryi* | 115.673 | 31.305 |
| *Emmenopterys henryi* | 115.681 | 29.620 |
| *Emmenopterys henryi* | 115.690 | 26.696 |
| *Emmenopterys henryi* | 115.708 | 31.209 |
| *Emmenopterys henryi* | 115.716 | 31.136 |
| *Emmenopterys henryi* | 115.742 | 31.322 |
| *Emmenopterys henryi* | 115.752 | 31.085 |
| *Emmenopterys henryi* | 115.760 | 31.171 |
| *Emmenopterys henryi* | 115.795 | 31.104 |
| *Emmenopterys henryi* | 115.800 | 29.410 |
| *Emmenopterys henryi* | 115.820 | 29.120 |
| *Emmenopterys henryi* | 115.844 | 29.435 |
| *Emmenopterys henryi* | 115.877 | 29.600 |
| *Emmenopterys henryi* | 115.879 | 31.303 |
| *Emmenopterys henryi* | 115.898 | 28.678 |
| *Emmenopterys henryi* | 115.900 | 29.400 |
| *Emmenopterys henryi* | 115.920 | 31.710 |
| *Emmenopterys henryi* | 115.925 | 29.579 |
| *Emmenopterys henryi* | 115.934 | 29.529 |
| *Emmenopterys henryi* | 115.965 | 31.166 |
| *Emmenopterys henryi* | 115.976 | 31.315 |
| *Emmenopterys henryi* | 115.977 | 29.548 |
| *Emmenopterys henryi* | 115.980 | 29.670 |
| *Emmenopterys henryi* | 115.984 | 29.451 |
| *Emmenopterys henryi* | 115.984 | 29.726 |
| *Emmenopterys henryi* | 115.986 | 29.598 |
| *Emmenopterys henryi* | 115.992 | 30.980 |
| *Emmenopterys henryi* | 116.046 | 30.979 |
| *Emmenopterys henryi* | 116.063 | 25.609 |
| *Emmenopterys henryi* | 116.201 | 31.130 |
| *Emmenopterys henryi* | 116.210 | 27.229 |
| *Emmenopterys henryi* | 116.213 | 31.222 |
| *Emmenopterys henryi* | 116.218 | 29.745 |
| *Emmenopterys henryi* | 116.220 | 27.540 |
| *Emmenopterys henryi* | 116.284 | 27.260 |
| *Emmenopterys henryi* | 116.320 | 26.830 |
| *Emmenopterys henryi* | 116.330 | 26.320 |
| *Emmenopterys henryi* | 116.332 | 31.284 |
| *Emmenopterys henryi* | 116.340 | 32.040 |
| *Emmenopterys henryi* | 116.348 | 25.997 |
| *Emmenopterys henryi* | 116.390 | 26.739 |
| *Emmenopterys henryi* | 116.450 | 26.283 |
| *Emmenopterys henryi* | 116.479 | 26.751 |
| *Emmenopterys henryi* | 116.772 | 31.305 |
| *Emmenopterys henryi* | 116.850 | 26.716 |
| *Emmenopterys henryi* | 116.930 | 31.460 |
| *Emmenopterys henryi* | 117.167 | 27.913 |
| *Emmenopterys henryi* | 117.424 | 27.995 |
| *Emmenopterys henryi* | 117.464 | 29.039 |
| *Emmenopterys henryi* | 117.540 | 29.624 |
| *Emmenopterys henryi* | 117.570 | 28.950 |
| *Emmenopterys henryi* | 117.582 | 27.937 |
| *Emmenopterys henryi* | 117.633 | 27.733 |
| *Emmenopterys henryi* | 117.682 | 28.847 |
| *Emmenopterys henryi* | 117.710 | 29.850 |
| *Emmenopterys henryi* | 117.733 | 27.816 |
| *Emmenopterys henryi* | 117.745 | 29.601 |
| *Emmenopterys henryi* | 117.746 | 29.543 |
| *Emmenopterys henryi* | 117.783 | 27.860 |
| *Emmenopterys henryi* | 118.026 | 28.931 |
| *Emmenopterys henryi* | 118.036 | 27.667 |
| *Emmenopterys henryi* | 118.042 | 29.554 |
| *Emmenopterys henryi* | 118.084 | 25.736 |
| *Emmenopterys henryi* | 118.116 | 29.066 |
| *Emmenopterys henryi* | 118.116 | 29.225 |
| *Emmenopterys henryi* | 118.129 | 30.295 |
| *Emmenopterys henryi* | 118.131 | 30.111 |
| *Emmenopterys henryi* | 118.159 | 30.176 |
| *Emmenopterys henryi* | 118.170 | 29.780 |
| *Emmenopterys henryi* | 118.179 | 29.603 |
| *Emmenopterys henryi* | 118.182 | 30.092 |
| *Emmenopterys henryi* | 118.208 | 29.442 |
| *Emmenopterys henryi* | 118.223 | 30.266 |
| *Emmenopterys henryi* | 118.227 | 25.652 |
| *Emmenopterys henryi* | 118.241 | 28.933 |
| *Emmenopterys henryi* | 118.283 | 29.433 |
| *Emmenopterys henryi* | 118.345 | 29.828 |
| *Emmenopterys henryi* | 118.446 | 28.063 |
| *Emmenopterys henryi* | 118.468 | 30.590 |
| *Emmenopterys henryi* | 118.472 | 28.405 |
| *Emmenopterys henryi* | 118.509 | 28.355 |
| *Emmenopterys henryi* | 118.548 | 30.576 |
| *Emmenopterys henryi* | 118.632 | 29.236 |
| *Emmenopterys henryi* | 118.648 | 29.314 |
| *Emmenopterys henryi* | 118.740 | 28.288 |
| *Emmenopterys henryi* | 118.783 | 30.023 |
| *Emmenopterys henryi* | 118.858 | 28.316 |
| *Emmenopterys henryi* | 118.862 | 30.101 |
| *Emmenopterys henryi* | 118.862 | 32.057 |
| *Emmenopterys henryi* | 118.883 | 28.366 |
| *Emmenopterys henryi* | 118.925 | 28.036 |
| *Emmenopterys henryi* | 118.931 | 30.035 |
| *Emmenopterys henryi* | 118.951 | 27.641 |
| *Emmenopterys henryi* | 118.983 | 30.033 |
| *Emmenopterys henryi* | 119.013 | 30.130 |
| *Emmenopterys henryi* | 119.023 | 25.993 |
| *Emmenopterys henryi* | 119.036 | 30.286 |
| *Emmenopterys henryi* | 119.049 | 30.021 |
| *Emmenopterys henryi* | 119.100 | 29.840 |
| *Emmenopterys henryi* | 119.100 | 30.180 |
| *Emmenopterys henryi* | 119.110 | 28.745 |
| *Emmenopterys henryi* | 119.111 | 30.619 |
| *Emmenopterys henryi* | 119.147 | 28.338 |
| *Emmenopterys henryi* | 119.154 | 27.901 |
| *Emmenopterys henryi* | 119.161 | 30.033 |
| *Emmenopterys henryi* | 119.166 | 30.110 |
| *Emmenopterys henryi* | 119.181 | 27.734 |
| *Emmenopterys henryi* | 119.221 | 30.169 |
| *Emmenopterys henryi* | 119.246 | 30.775 |
| *Emmenopterys henryi* | 119.256 | 31.087 |
| *Emmenopterys henryi* | 119.288 | 29.443 |
| *Emmenopterys henryi* | 119.313 | 28.073 |
| *Emmenopterys henryi* | 119.360 | 30.190 |
| *Emmenopterys henryi* | 119.375 | 31.417 |
| *Emmenopterys henryi* | 119.390 | 29.980 |
| *Emmenopterys henryi* | 119.400 | 30.300 |
| *Emmenopterys henryi* | 119.410 | 30.890 |
| *Emmenopterys henryi* | 119.412 | 29.420 |
| *Emmenopterys henryi* | 119.414 | 30.410 |
| *Emmenopterys henryi* | 119.424 | 30.349 |
| *Emmenopterys henryi* | 119.430 | 29.648 |
| *Emmenopterys henryi* | 119.433 | 28.416 |
| *Emmenopterys henryi* | 119.446 | 28.732 |
| *Emmenopterys henryi* | 119.470 | 30.260 |
| *Emmenopterys henryi* | 119.470 | 31.410 |
| *Emmenopterys henryi* | 119.483 | 31.270 |
| *Emmenopterys henryi* | 119.502 | 31.180 |
| *Emmenopterys henryi* | 119.516 | 30.349 |
| *Emmenopterys henryi* | 119.610 | 31.140 |
| *Emmenopterys henryi* | 119.618 | 30.263 |
| *Emmenopterys henryi* | 119.639 | 27.970 |
| *Emmenopterys henryi* | 119.645 | 29.651 |
| *Emmenopterys henryi* | 119.650 | 27.700 |
| *Emmenopterys henryi* | 119.700 | 30.258 |
| *Emmenopterys henryi* | 119.710 | 30.095 |
| *Emmenopterys henryi* | 119.722 | 28.868 |
| *Emmenopterys henryi* | 119.738 | 31.194 |
| *Emmenopterys henryi* | 119.739 | 27.607 |
| *Emmenopterys henryi* | 119.742 | 30.488 |
| *Emmenopterys henryi* | 119.742 | 31.257 |
| *Emmenopterys henryi* | 119.747 | 30.259 |
| *Emmenopterys henryi* | 119.781 | 28.187 |
| *Emmenopterys henryi* | 119.798 | 28.018 |
| *Emmenopterys henryi* | 119.820 | 31.370 |
| *Emmenopterys henryi* | 119.822 | 30.235 |
| *Emmenopterys henryi* | 119.904 | 31.522 |
| *Emmenopterys henryi* | 119.908 | 27.775 |
| *Emmenopterys henryi* | 119.910 | 30.030 |
| *Emmenopterys henryi* | 119.933 | 28.416 |
| *Emmenopterys henryi* | 119.960 | 28.786 |
| *Emmenopterys henryi* | 119.986 | 29.106 |
| *Emmenopterys henryi* | 120.019 | 29.647 |
| *Emmenopterys henryi* | 120.155 | 30.268 |
| *Emmenopterys henryi* | 120.204 | 30.331 |
| *Emmenopterys henryi* | 120.446 | 28.854 |
| *Emmenopterys henryi* | 120.508 | 28.983 |
| *Emmenopterys henryi* | 120.573 | 29.992 |
| *Emmenopterys henryi* | 120.748 | 28.979 |
| *Emmenopterys henryi* | 120.766 | 28.515 |
| *Emmenopterys henryi* | 120.910 | 29.233 |
| *Emmenopterys henryi* | 120.916 | 28.816 |
| *Emmenopterys henryi* | 121.022 | 28.992 |
| *Emmenopterys henryi* | 121.100 | 29.250 |
| *Emmenopterys henryi* | 121.769 | 29.790 |
